# Additional Sex Combs-Like 2 Is Required for Polycomb Repressive Complex 2 Binding at Select Targets

**DOI:** 10.1371/journal.pone.0073983

**Published:** 2013-09-09

**Authors:** Hsiao-Lei Lai, Q. Tian Wang

**Affiliations:** Department of Biological Sciences, University of Illinois at Chicago, Chicago, Illinois, United States of America; University of Hawaii at Manoa, John A. Burns School of Medicine, United States of America

## Abstract

Polycomb Group (PcG) proteins are epigenetic repressors of gene expression. The Drosophila *Additional sex combs* (*Asx*) gene and its mammalian homologs exhibit PcG function in genetic assays; however, the mechanism by which Asx family proteins mediate gene repression is not well understood. ASXL2, one of three mammalian homologs for Asx, is highly expressed in the mammalian heart and is required for the maintenance of cardiac function. We have previously shown that *Asxl2* deficiency results in a reduction in the bulk level of histone H3 lysine 27 trimethylation (H3K27me3), a repressive mark generated by the Polycomb Repressive Complex 2 (PRC2). Here we identify several ASXL2 target genes in the heart and investigate the mechanism by which ASXL2 facilitates their repression. We show that the *Asxl2-*deficient heart is defective in converting H3K27me2 to H3K27me3 and in removing ubiquitin from mono-ubiquitinated histone H2A. ASXL2 and PRC2 interact in the adult heart and co-localize to target promoters. ASXL2 is required for the binding of PRC2 and for the enrichment of H3K27me3 at target promoters. These results add a new perspective to our understanding of the mechanisms that regulate PcG activity and gene repression.

## Introduction

During the development and life of multicellular organisms, there is a need to set up and maintain distinct identities in different types of cells and tissues. Epigenetic mechanisms play critical roles in the establishment and maintenance of cellular identity. Polycomb Group (PcG) proteins were originally identified in Drosophila as repressors of homeotic genes (*Hox* genes) [1]. The balanced action of PcG proteins and their antagonists, the Trithorax Group (TrxG) epigenetic activators, is crucial for the maintenance of *Hox* expression domains along the anterior–posterior axis [1,2]. It has since been discovered that PcG and TrxG proteins play essential roles in mammalian development, regulating the differentiation of a wide array of cell lineages [3–5].

PcG proteins form multi-subunit complexes and function at the level of chromatin. One of the best characterized PcG complexes is the Polycomb Repressive Complex 2 (PRC2). PRC2 is responsible for generation of histone H3 lysine 27 trimethylation (H3K27me3), a mark that is associated with a silent chromatin state [6,7]. The core components of PRC2, EZH2, SUZ12 and EED, are necessary and sufficient for PRC2’s histone methyltransferase (HMTase) activity [7–10]. The SET-domain protein EZH2 is the catalytic subunit [6,7]. SUZ12 is required for the integrity of PRC2 and for preventing proteolytic degradation of EZH2 [8,10]. EED binds to H3 tails carrying trimethylated K27 and stimulates the HMTase activity of EZH2, thereby facilitating the spread of the H3K27me3 mark to neighboring nucleosomes [11].

The Drosophila *Additional sex combs* (*Asx*) gene was initially identified based on PcG-like mutant phenotypes and genetic interaction with other PcG genes [12,13]. Recently, Asx was shown to associate with the histone deubiquitinase Calypso to form the Polycomb Repressive Deubiquitinase (PR–DUB) complex [14]. Asx plays at least two roles in the PR–DUB complex: to stabilize the Calypso protein and to stimulate its deubiquitinase activity, which is specific for mono-ubiquitinated histone H2A (uH2A). The deubiquitinase activity of PR–DUB is required for repression of *Ubx* in Drosophila wing disc. These results provided important insight into the biological function of Asx and PR–DUB. However, it remains unclear how uH2A deubiquitination contributes to the repression of PcG target genes.

There are three Asx homologs in human and mouse genomes, *Asx-like 1* (*Asxl1*), *Asxl2* and *Asxl3*

[16-18]. We have previously generated an *Asxl2* mutant mouse strain, which carries a gene-trapped allele that severely reduces *Asxl2* expression [19]. Initial characterization of *Asxl2* ^*-/-*^ mice has confirmed functional conservation between *Asxl2* and *Asx* and has shown that *Asxl2* is highly expressed in the heart [19]. Interestingly, *Asxl2* ^*-/-*^ hearts exhibit significant reduction in the level of bulk H3K27me3, suggesting that ASXL2 regulates PRC2 activity [19]. Here we explore the molecular basis by which ASXL2 mediates gene repression in the heart.

## Results

### Asxl2 is associated with chromatin

**Figure 1:**
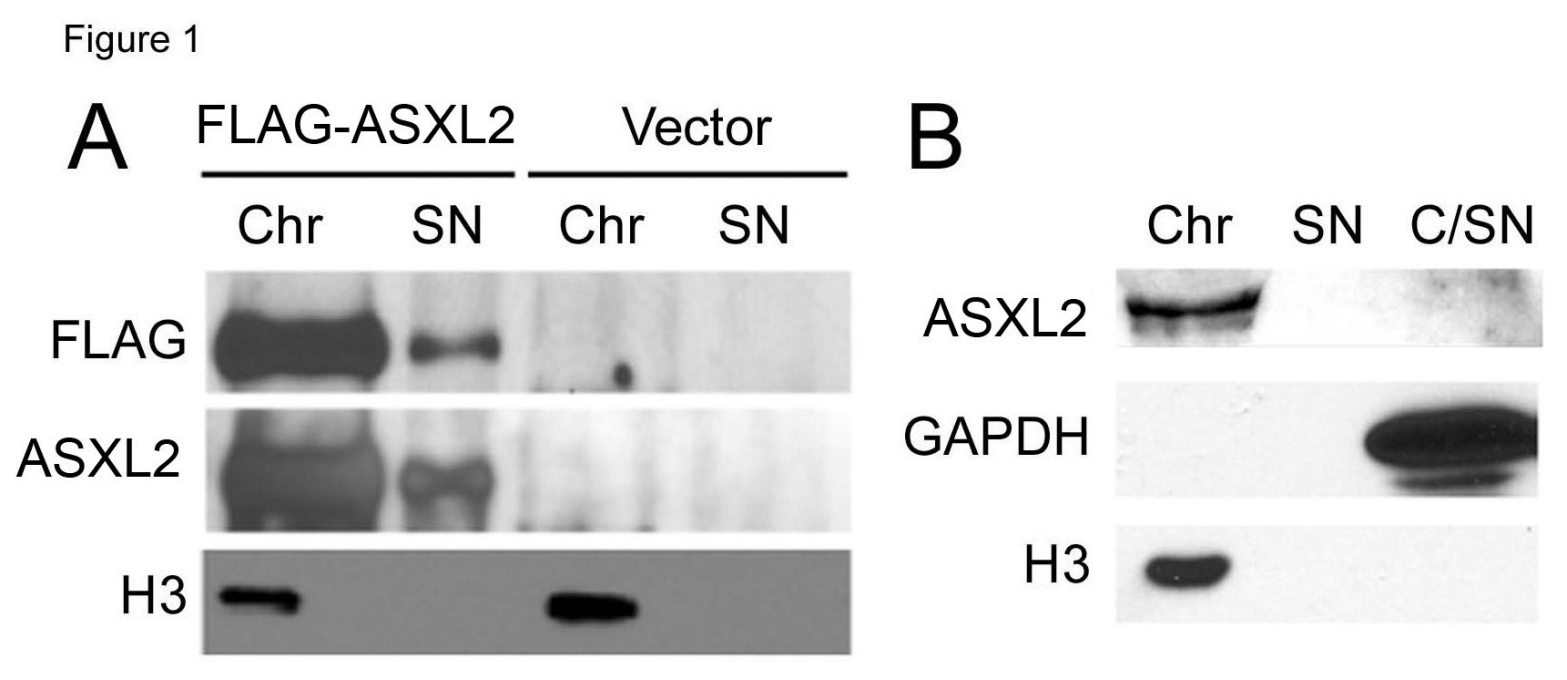
ASXL2 is associated with chromatin. (A) FLAG-ASXL2 is associated with chromatin in transfected HEK293 cells. Biochemical fractions were prepared from HEK293 cells transfected with either FLAG-ASXL2 or vector. Western blot assays were performed using M2 anti-FLAG antibody and KC17 anti-ASXL2 antibody, respectively. Each lane contains 10% of the indicated fraction. An anti-histone H3 antibody (Active Motif) was used to confirm the quality of fractionation. (B) Endogenous ASXL2 is associated with chromatin. Biochemical fractions were prepared from heart tissue and probed with KC17 antibody. Each lane contains 3% of the indicated fraction. Anti-GAPDH (Millipore) and anti-histone H3 antibodies were used to confirm the quality of fractionation. Chr: chromatin fraction. SN: soluble nuclear fraction. C/SN: cytosol fraction with trace soluble nuclear proteins.

Drosophila Asx is a chromatin-associated protein [15]. Immunostaining of polytene chromosomes identified 90 Asx binding sites, ~70% of which overlapped with binding sites of other PcG proteins [15]. A recent ChIP-on-chip study identified 879 PR–DUB binding sites with high confidence in the Drosophila genome [14]. To confirm that murine ASXL2 is also associated with chromatin, we expressed FLAG-tagged ASXL2 in HEK293 cells and used biochemical fractionation [20] to separate chromatin-associated proteins from soluble nuclear proteins. Probing the fractions with either the anti-ASXL2 antibody KC17 [21] or with anti-FLAG antibody M2 (Sigma) detected ASXL2 predominantly in the chromatin fraction (Figure 1A). Similar results were obtained with endogenous ASXL2 in murine heart tissue (Figure 1B).

### Asxl2 is required for the normal expression of multiple cardiac genes

**Figure 2:**
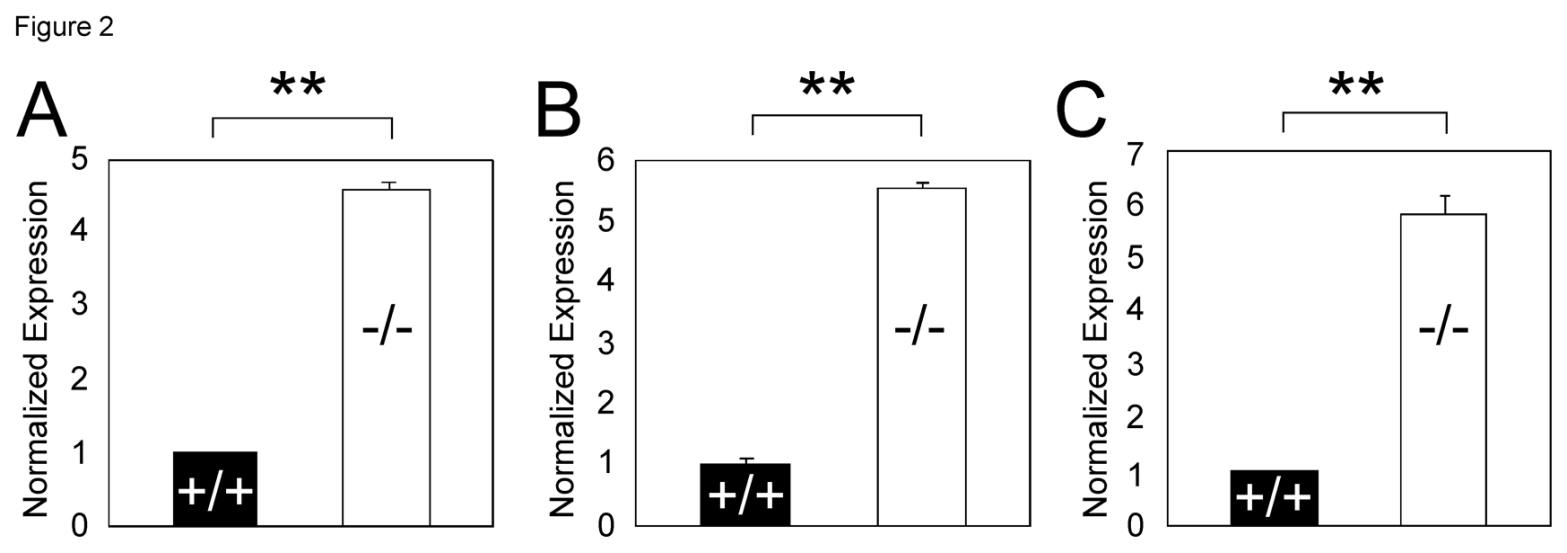
ASXL2 is required for the repression of select cardiac genes. The mRNA levels of *Sfrp2* (A), *Acta1* (B), and *Grk5* (C) in wild-type and *Asxl2* ^*-/-*^ hearts were analyzed by real-time RT-PCR. Each column shown is the mean value of data generated from three independent samples. **p<0.01; Error bar: standard deviation.

We have recently shown that ASXL2 is required for the long-term maintenance of cardiac function in adult mice [21]. The loss of cardiac function in *Asxl2* ^*-/-*^ hearts is correlated with de-repression of myosin heavy chain β (β-MHC), the fetal form of MHC that has lower ATPase activity than the adult alpha form [21]. We showed that ASXL2 and the PRC2 core component EZH2 co-localized to multiple conserved regions within the β*-MHC* promoter. This, along with our previous observation that the level of bulk H3K27me3 is significantly reduced in *Asxl2* ^*-/-*^ hearts, led us to hypothesize that ASXL2 and PRC2 may act together to regulate the expression of β*-MHC* and other target genes. To investigate this hypothesis, we first sought to identify additional targets of ASXL2 in the murine heart. We performed a microarray analysis on 1-month-old wild-type and *Asxl2* ^*-/-*^ hearts and identified 753 genes that are either induced or repressed more than 2 fold in *Asxl2* ^*-/-*^ hearts (Table S1). The mis-expression of these genes is unlikely a secondary effect due to cardiac stress, because ventricular function is largely normal in *Asxl2* ^*-/-*^ hearts at this early stage [21]. We chose to examine three genes, in addition to β*-MHC*, in more detail: *Secreted frizzled-related protein 2* (*Sfrp2*); *Actin,* alpha *1, skeletal muscle* (*Acta1*); and *Gprotein-coupled* *receptor kinase 5* (*Grk5*). First, query of the Broad Institute ChIP-seq database revealed that the promoters of these genes are enriched for PRC2 components and H3K27me3 in embryonic stem (ES) cells (Fig. S1). This suggests that these loci contain regulatory elements needed to recruit PcG activity. Therefore, they are good candidates as PcG target genes in not only ES cells but also in differentiated cells/tissues, including the heart. In fact, *Sfrp2* has been shown to be a PcG target in human embryonic fibroblasts [22]. Second, all three genes have been implicated in congenital or acquired heart diseases/conditions in human and/or mouse [23–26], suggesting that an understanding of their regulation could be clinically important. Using real-time RT-PCR, we confirmed that *Sfrp2*, *Acta1* and *Grk5* are de-repressed in *Asxl2* ^*-/-*^ hearts by 4.6, 5.8, and 5.9 folds, respectively (Figure 2).

### ASXL2 and PRC2 components co-localize at select target loci

**Figure 3:**
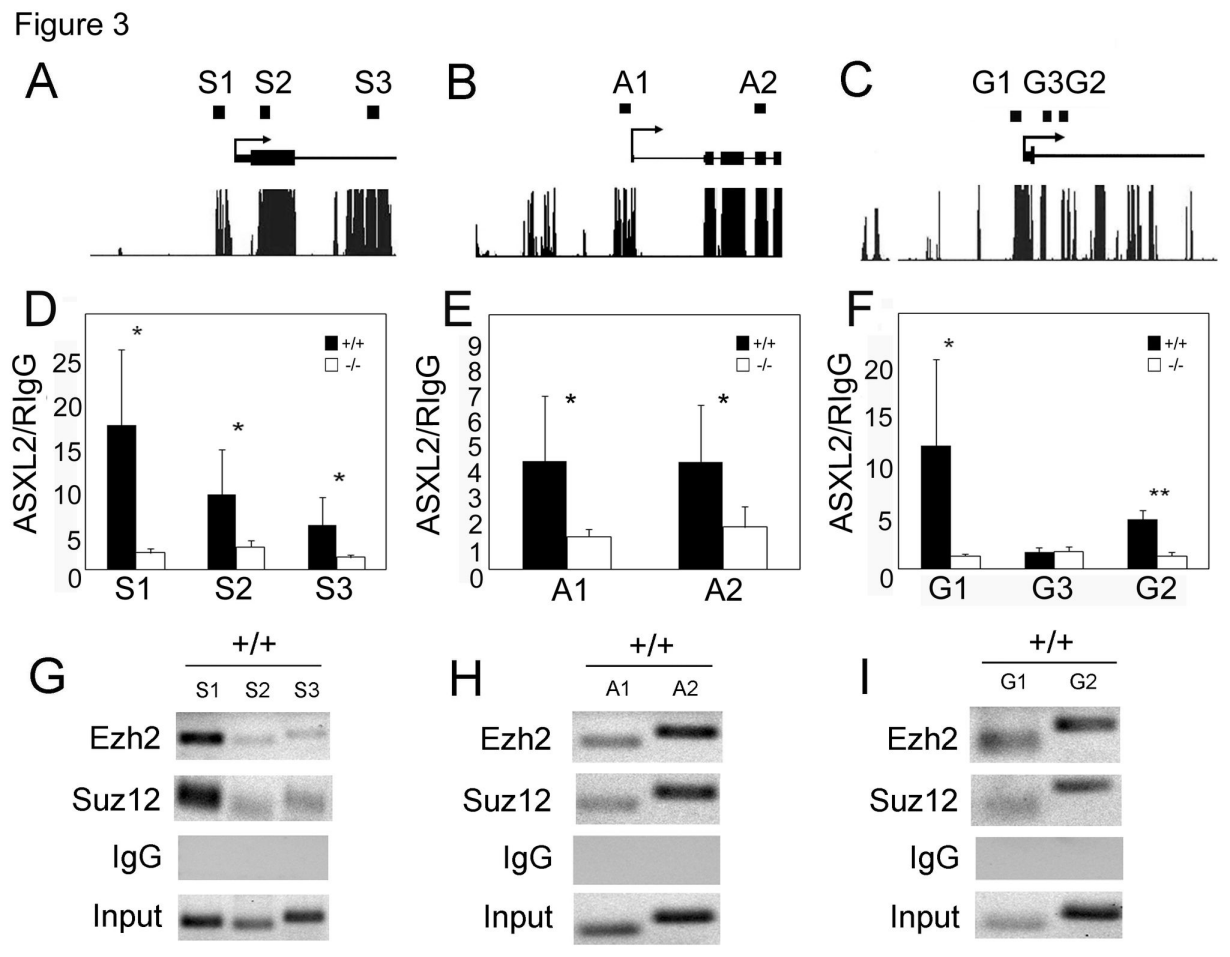
ASXL2 and PRC2 core components co-localize at select target loci. (A–C) Alignment of mouse, rat and human genomic sequences from -2kb to +2kb of *Sfrp2* (A), *Acta1* (B), and *Grk5* (C). The peaks correspond to regions of sequence conservation. For each gene, 2-3 highly conserved regions (black bars on top of the graphs, designated S1-3, A1-2 and G1-3, respectively) were selected for ChIP analysis. (D–F) ChIP-qPCR assays of ASXL2 enrichment near *Sfrp2* (D), *Acta1* (E) and *Grk5* (F) TSSs in 1-month-old wild-type and *Asxl2* ^*-/-*^ hearts. Each column represents the mean value of data from three independent samples. Mock ChIPs were performed with rabbit IgG. (G–I) ChIP-PCR assays of EZH2 and SUZ12 enrichment near *Sfrp2* (G), *Acta1* (H) and *Grk5* (I) TSSs in 1-month-old wild-type mouse hearts. Input: PCR assays of 1:100 diluted total input chromatin. *p<0.05; **p<0.01; Error bar: standard deviation.

Genome-wide studies have consistently found PRC2 components to be enriched at chromatin regions near the transcription start sites (TSSs) of target genes [27–34]. To determine whether *Sfrp2*, *Acta1* and *Grk5* are directly repressed by ASXL2 and PRC2, we examined enrichment of ASXL2 and PRC2 components at these loci by ChIP-qPCR assays, focusing on regions between -2 kb and +2 kb of the TSS. For each locus, we selected 2-3 genomic sites that are conserved between mouse, rat and human (Figure 3A–C). ASXL2 was enriched at most of these sites (Figure 3D–F). Most of the ASXL2-enriched sites also exhibited enrichment of PRC2 core components EZH2 and SUZ12 (Figure 3G–I).

**Figure 4:**
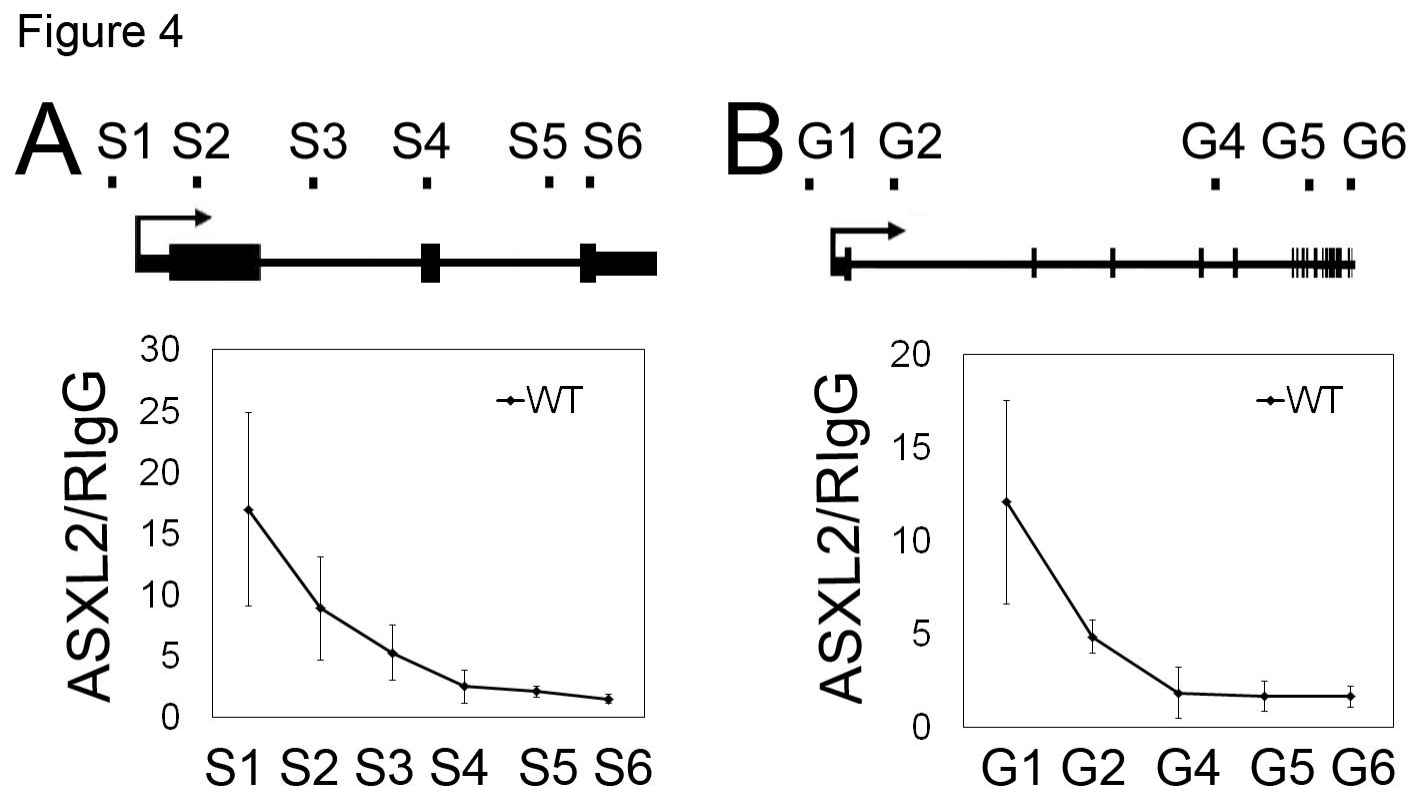
Distribution of ASXL2 along *Sfrp2* (A) and *Grk5* (B) loci. For each gene, the level of ASXL2 enrichment was analyzed by ChIP-qPCR at multiple conserved regions distributed between the promoter and the 3’ UTR. Mock ChIPs were performed with rabbit IgG. Each column represents the mean value of data from three independent samples. Error bar: standard deviation.

To investigate the distribution of ASXL2 along target loci, we selected a series of conserved sites within the gene bodies of *Sfrp2* and *Grk5* and examined the level of ASXL2 enrichment by ChIP-qPCR assays. For both genes, ASXL2 was most highly enriched at the promoter, and the level of enrichment decreases from 5’ to 3’ of the gene (Figure 4A-B).

To confirm that we are detecting site-specific binding of ASXL2 instead of promiscuous binding to chromatin, ChIP assays were also performed for the *S100a10* locus, which was active in both wild-type and *Asxl2* ^*-/-*^ hearts. ASXL2 enrichment was not detected at any of the six sites that we analyzed for the *S100a10* locus (Figure S2).

### H3K27me3 is significantly reduced at de-repressed ASXL2 target loci

**Figure 5:**
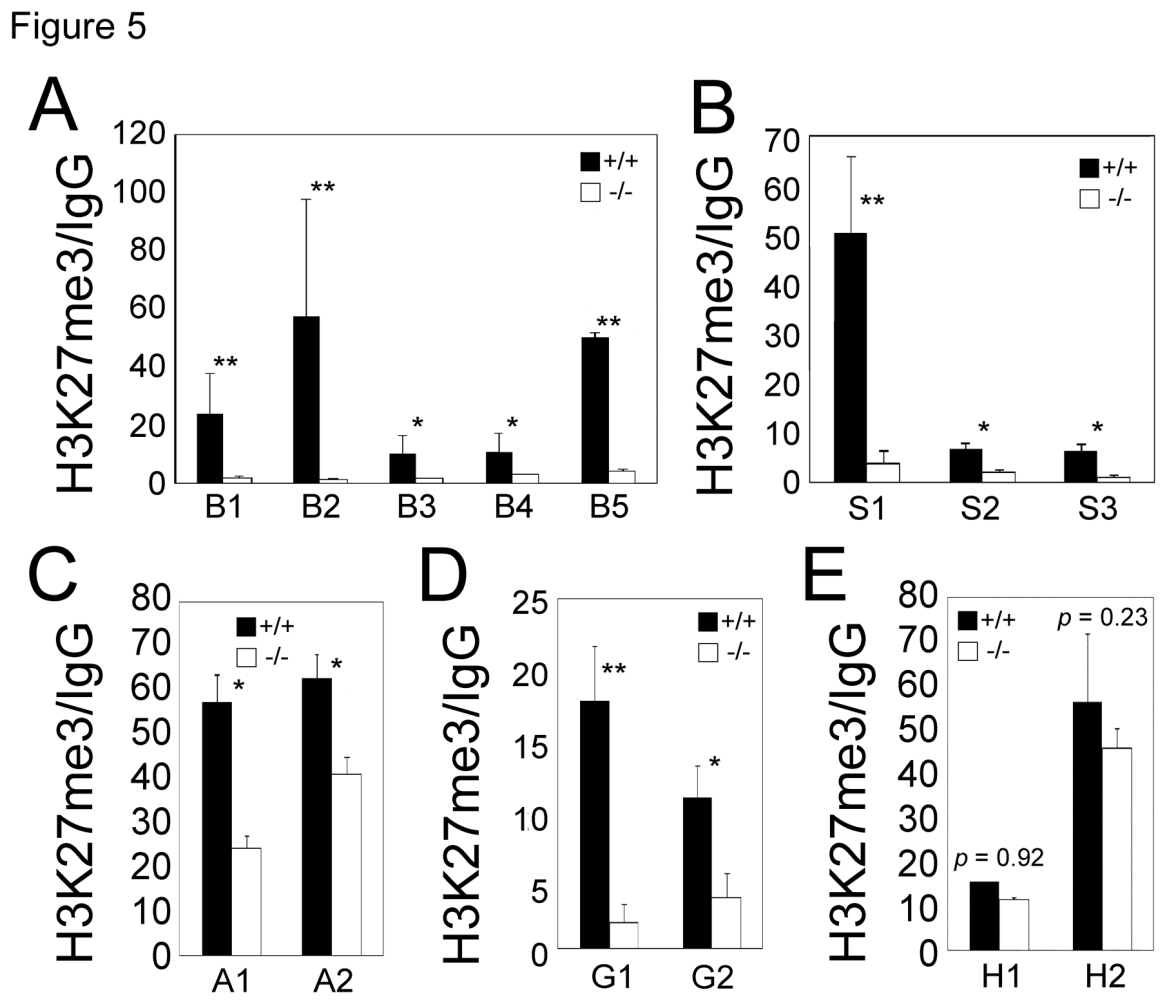
ChIP-qPCR assays of H3K27me3 enrichment at β*-MHC* (A), *Sfrp2* (B), *Acta1* (C), *Grk5* (D) and *Hoxb5* (E) loci in wild-type and *Asxl2* ^*-/-*^ hearts. Data from H3K27me3 ChIP were normalized against those from IgG mock ChIP. Each column represents the mean value of data from three independent samples. The five conserved regions in the β*-MHC* promoter, B1-5, are as previously described (79). Genomic positions of H1 and H2 within the *Hoxb5* locus are shown in Figure S4. *p<0.05; **p<0.01; Error bar: standard deviation.

We have previously shown that the bulk level of H3K27me3 is decreased in *Asxl2* ^*-/-*^ hearts [19]. This is consistent with genetic evidence in both Drosophila and mouse suggesting that *Asx* and *Asx-like* genes promote PcG activity [19,35,36]. We hypothesized that de-repression of β*-MHC*, *Sfrp2, Acta1* and *Grk5* in the *Asxl2* ^*-/-*^ heart is due to a deficiency of H3K27me3 at these loci. ChIP-qPCR assay showed that in comparison to wild-type hearts, *Asxl2* ^*-/-*^ hearts exhibited significant reductions in the level of H3K27me3 enrichment at β*-MHC*, *Sfrp2, Acta1* and *Grk5* promoters (Figure 5A–D, Figure S3), confirming our hypothesis. In contrast, the level of H3K27me3 enrichment at the *Hoxb5* locus did not change in *Asxl2* ^*-/-*^ hearts (Figure 5E, Figure S4). Additionally, qRT-PCR detected extremely low, if any, *Hoxb5* transcription in both wild-type and *Asxl2* ^*-/-*^ hearts (data not shown), suggesting that it does not require ASXL2 for repression. These results suggest that ASXL2 is specifically involved in the regulation of a subset of PcG targets.

### Acetylation of histone H3 (AcH3) is significantly increased at de-repressed ASXL2 target loci

**Figure 6:**
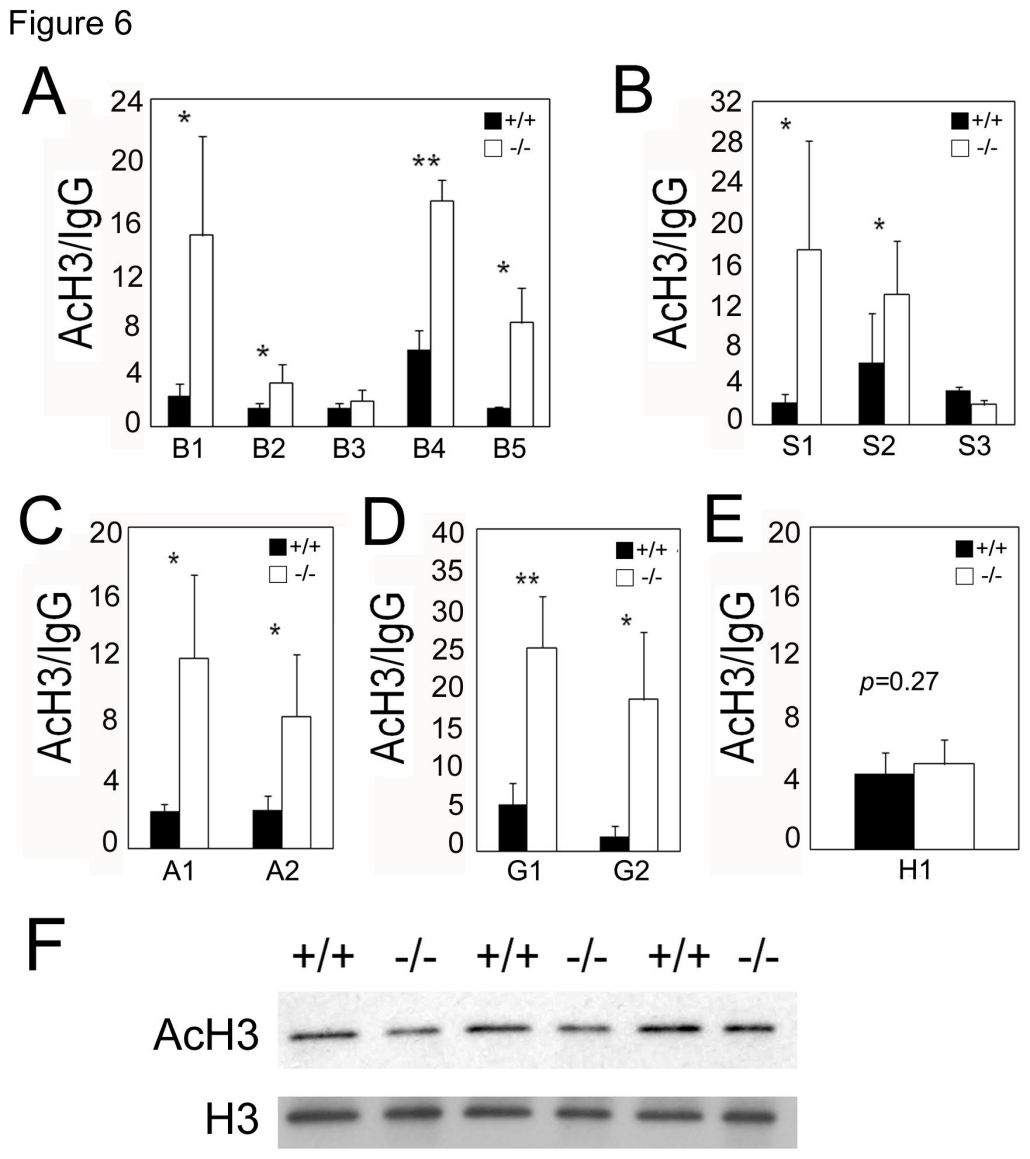
ChIP-qPCR assays of AcH3 enrichment at β*-MHC* (A), *Sfrp2* (B), *Acta1* (C), *Grk5* (D) and *Hoxb5* (E) loci in wild-type and *Asxl2* ^*-/-*^ hearts. Data from AcH3 ChIP were normalized against those from IgG mock ChIP. Each column represents the mean value of data from three independent samples. *p<0.05; **p<0.01; Error bar: standard deviation. (F) Western blot analysis of bulk AcH3 in three pairs of wild-type and *Asxl2* ^*-/-*^ hearts. To control for comparable protein loading, the blot was stripped and re-blotted for histone H3.

To test the possibility that the loss of *Asxl2* may result in depletion of nucleosomes or indiscriminate reduction of all histone modifications at target loci, we examined the enrichment of AcH3, an active histone mark [37]. In the absence of *Asxl2*, the level of AcH3 enrichment increased significantly at β*-MHC, Sfrp2, Acta1 and Grk5* – loci that are dependent on ASXL2 for repression (Figure 6A–D). No increase of AcH3 was observed at the *Hoxb5* locus, which does not require ASXL2 for repression (Figure 6E). The bulk level of AcH3 is comparable in wild-type and *Asxl2* ^*-/-*^ hearts (Figure 6F). Taken together, *Asxl2* deficiency specifically affects H3K27 methylation.

### PRC2 core subunits are expressed and form complexes in Asxl2^-/-^ hearts

To understand the mechanism by which ASXL2 regulates H3K27me3 levels at target chromatin loci, we first asked whether

ASXL2 is required for the stability of PRC2 core subunits. Nuclear protein extracts from wild-type and

**Figure 7:**
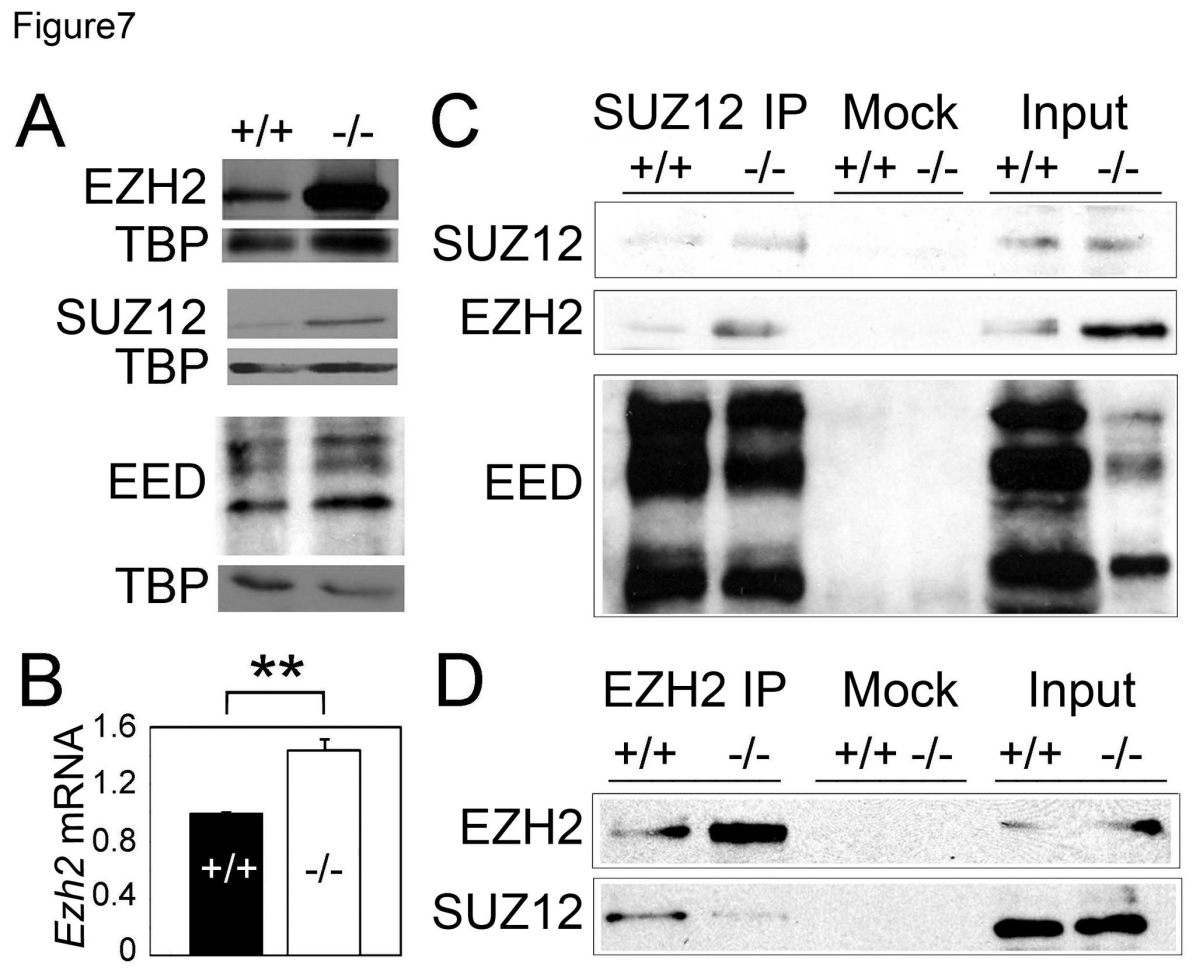
ASXL2 is not required for the protein stability of PRC2 core components or the integrity of PRC2 complex. (A) Western blot analysis of protein levels of EZH2, SUZ12, and EED in wild-type and *Asxl2* ^*-/-*^ hearts. Western blot of TATA-binding protein (TBP) was used as a loading control. Three pairs of hearts were analyzed and a representative result was shown for each protein. (B) Real-time RT-PCR analysis of *Ezh2* transcripts in wild-type and *Asxl2* ^*-/-*^ hearts. **p<0.01; Error bar: standard deviation. (C, D) Co-IP analysis of interaction between PRC2 components. Wild-type and *Asxl2* ^*-/-*^ heart extracts were IPed using either an anti-SUZ12 antibody (C) or an anti-EZH2 antibody (D). Mock IP was performed with pre-immune serum. IPed samples were analyzed by Western blot using the indicated antibodies.

*Asxl2* ^*-/-*^ hearts were separated on SDS-PAGE and probed with antibodies against EZH2, SUZ12, and EED (Figure 7A). The level of EZH2 protein is increased by approximately 2.6-fold in *Asxl2* ^*-/-*^ hearts compared to that in wild-type (Figure S5). The levels of SUZ12 and EED also increased but to lesser degrees. Real-time RT-PCR showed that transcription of EZH2 is increased by 1.4-fold in *Asxl2* ^*-/-*^ hearts (Figure 7B). Taken together, these results suggest that *Asxl2* is not required for the expression of EZH2, SUZ12 or EED. Instead, the loss of *Asxl2* and the subsequent reduction in bulk H3K27me3 may have triggered a compensation pathway to express more PRC2 components.

Next, we asked whether deficiency in *Asxl2* affects the association between PRC2 core components. We immunoprecipitated SUZ12 and proteins associated with it from wild-type and *Asxl2* ^*-/-*^ heart extracts. Western blot analysis showed that EZH2 and EED co-IPed with SUZ12 in both wild-type and *Asxl2* ^*-/-*^ hearts (Figure 7C). In addition, immunoprecipitation of EZH2 pulled down SUZ12 (Figure 7D). These results suggest that *Asxl2* is dispensable for the formation of the PRC2 core complex.

### Asxl2 is required for PRC2 binding at target loci

**Figure 8:**
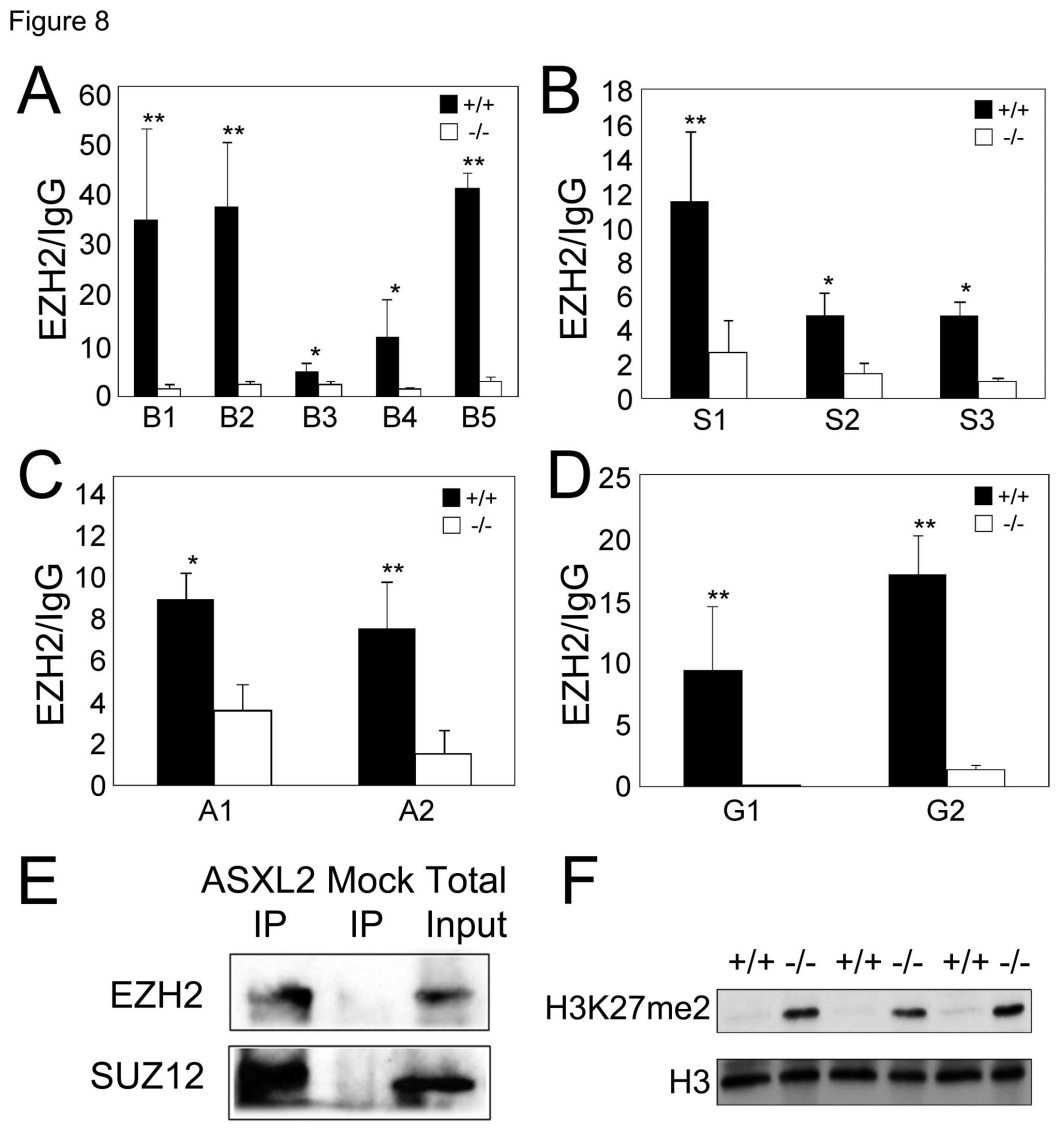
ASXL2 interacts with PRC2 and is required for PRC2 enrichment at select target genes in the mouse heart. The level of EZH2 enrichment at β*-MHC* (A), *Sfrp2* (B), *Acta1* (C) and *Grk5* (D) in wild-type and *Asxl2* ^*-/-*^ hearts was compared by ChIP-qPCR. Data from EZH2 ChIP were normalized against those from IgG mock ChIP. Each column represents the mean value of data from three independent samples. *p<0.05; **p<0.01; Error bar: standard deviation. (E) Co-IP analysis of the interaction between ASXL2 and PRC2 components. Wild-type heart extract was IPed using KC17 anti-ASXL2 antibody. Mock IP was performed with pre-immune rabbit serum. IPed samples were analyzed by Western blot using anti-EZH2 and anti-SUZ12. (F) Western blot analysis of bulk H3K27me2 in three pairs of wild-type and *Asxl2* ^*-/-*^ hearts. To control for comparable protein loading, the blot was stripped and re-blotted for histone H3.

Next, we asked whether ASXL2 plays a role in the localization of PRC2 to target chromatin. We compared the level of EZH2 enrichment at β*-MHC*, *Sfrp2, Acta1* and *Grk5* loci in wild-type and *Asxl2* ^*-/-*^ hearts by ChIP-qPCR. For all the sites that exhibited EZH2 enrichment above background in wild-type hearts, there is a significant reduction in chromatin-bound EZH2 in *Asxl2* ^*-/-*^ hearts (Figure 8A–D, Figure S6). Therefore, although *Asxl2* ^*-/-*^ hearts expressed a much higher level of EZH2 protein (Figure 7A), it failed to bind to ASXL2 target loci, which may account for reduced H3K27me3 levels at these loci and thereby de-repression.

### ASXL2 interacts with PRC2 core components in the adult heart

Given that ASXL2 co-localizes with PRC2 at target loci and is required for PRC2 binding, we tested whether ASXL2 interacts with PRC2 *in vivo*. We immunoprecipitated ASXL2 from heart extracts and examined the presence of EZH2 and SUZ12. As shown in Figure 8E, both PRC2 core components co-IPed with ASXL2. This suggests that ASXL2 associates with PRC2 in the heart and may regulate chromatin binding of PRC2 directly.

### Asxl2 is specifically required for the addition of the third methyl group to H3K27

PRC2 mediates the mono-, di- and tri- methylation of H3K27. It has been proposed that a stable association of PRC2 with chromatin is specifically required for the conversion of H3K27me2 to H3K27me3 [38]. Since the loss of *Asxl2* resulted in a significant decrease in the bulk level of H3K27me3 [19] and, at the same time, a decrease in PRC2 association with target loci (Figure 8A–D), we asked whether ASXL2 is specifically required for the addition of the third methyl group. Western blot analysis showed a striking increase in the level of bulk H3K27me2 in *Asxl2* ^*-/-*^ hearts (Figure 8F). This further confirms that PRC2 complex is intact and enzymatically active but fails to stably associate with chromatin in the absence of ASXL2.

### ASXL2 interacts with BAP1 in vivo and is required for efficient uH2A deubiquitination

**Figure 9:**
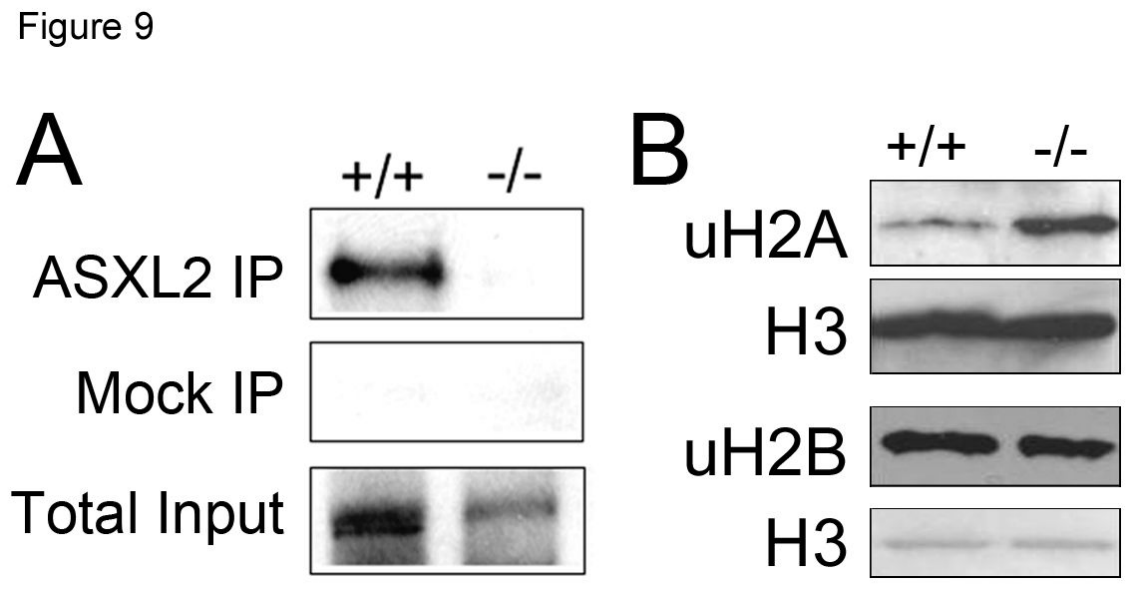
ASXL2 interacts with BAP1 *in vivo* and is required for efficient deubiquitination of uH2A. (A) Co-IP analysis of interaction between ASXL2 and BAP1. Wild-type and *Asxl2* ^*-/-*^ heart extracts were IPed using KC17 anti-ASXL2 antibody. Mock IP was performed with pre-immune rabbit serum. IPed samples were run on SDS-PAGE and probed with an anti-BAP1 antibody (Millipore). (B) Western blot analysis of bulk uH2A and uH2B in wild-type and *Asxl2* ^*-/-*^ hearts. To control for comparable protein loading, the blot was stripped and re-blotted for histone H3. The results shown are representative of three sets of experiments, each using a pair of wild-type and *Asxl2* ^*-/-*^ hearts.

Drosophila Asx is a component of the PR–DUB complex and is required for efficient deubiquitination of uH2A [14]. To determine whether this function is conserved in ASXL2, we examined the interaction between ASXL2 and BAP1, the mammalian homolog of Calypso, and the effect of *Asxl2* deficiency on bulk uH2A level. We found that BAP1 co-IPed with ASXL2 from wild-type heart extract (Figure 9A). In addition, the level of bulk uH2A was significantly increased in *Asxl2* ^*-/-*^ hearts (Figure 9B). The level of bulk uH2B did not change, consistent with previous report that PR–DUB specifically deubiquitinates uH2A but not uH2B [14]. These results suggest that ASXL2 is a critical component of mammalian PR–DUB in the heart.

## Discussion

### ASXL2 regulates PRC2-chromatin association

We have previously observed that the level of bulk H3K27me3 was reduced in *Asxl2* ^*-/-*^ hearts, suggesting an important role for ASXL2 in the homeostasis of the H3K27me3 mark [19]. H3K27 methylation is catalyzed by PRC2. PRC2 alone is sufficient for the mono- and di- methylation of H3K27 both *in vitro* and *in vivo* [39], and it has been proposed that H3K27 di-methylation may be accomplished prior to histone deposition [38]. On the other hand, efficient conversion of H3K27me2 to H3K27me3 is thought to require stable association of PRC2 with target chromatin [38,39].

Here we show that ASXL2 co-IPs with PRC2 and co-localizes with PRC2 at selected target loci. The loss of *Asxl2* results in loss of H3K27me3 enrichment at target promoters and gene de-repression. Further investigation showed that *Asxl2* deficiency did not reduce the expression of PRC2 components or prevent the formation of PRC2 complex, but specifically affected the association of PRC2 complex with target chromatin. Consistent with a requirement for ASXL2 in PRC2 binding to chromatin, *Asxl2* ^*-/-*^ hearts exhibit a significant increase in the level of bulk H3K27me2. Taken together, these results strongly suggest that ASXL2 is a regulator of PRC2-chromatin association and specifically promotes the addition of the third methyl group to H3K27.

A recent paper has shown that ASXL1 is required for PRC2 binding at target loci in human hematopoietic cells [40], suggesting that it is a conserved function of ASXL proteins. Like ASXL2 in the heart, ASXL1 is required in hematopoietic cells for maintaining the normal level of bulk H3K27me3. It will be interesting to determine whether there is an increase in H3K27me2 in *ASXL1* deficient blood cells. While the functional mechanism of ASXL1 and 2 may be similar, the two proteins are expressed in different tissues and have different target genes. *Asxl2* is the only *Asxl* gene that is highly expressed in the heart, and *Asxl2* ^*-/-*^ hearts did not exhibit up-regulation of either *Asxl1* or *Asxl3* (Figure S7). ASXL1 is required for the enrichment of PRC2 and H3K27me3 at the *HOXA* gene cluster in the hematopoietic lineage [40]. In the absence of *ASXL1*, *HOXA* genes are de-repressed. In contrast, ASXL2 appears dispensable for *Hox* gene repression in the heart (Table S1); the loss of *Asxl2* did not disrupt PRC2 and H3K27me3 enrichment at the *Hoxb5* locus (Figure 5E, Figure 6E, Figure S4). What could account for this difference? We propose that ASXL proteins are general facilitators of PRC2 recruitment and through their interaction with additional partners, such as transcription factors, target specificity in a given tissue can be achieved.

### ASXL2 and PHF1 use different mechanisms to promote H3K27 trimethylation

The function of ASXL2 in promoting H3K27 trimethylation is reminiscent of PHF1 (also known as PCL1), which interacts with EZH2 [38,41,42] and is essential for converting H3K27me2 to H3K27me3 at target loci [38,39]. However, there are three important distinctions.

First, PHF1 can be an integral component of PRC2 and co-purifies with the core components [38,39,42]. Although ASXL2 co-IPs with PRC2 from heart extract, neither Asx nor any ASXL proteins have been found to be part of PRC2. The interaction between ASXL2 and PRC2 may be indirect.

Secondly, PHF1 deficiency did not affect the level of bulk H3K27me2 or H3K27me3 [39]. Thus, ASXL2 appears to play a broader role than PHF1 in the regulation of PRC2. One possible scenario is that different genes require different proteins for the promotion of H3K27 trimethylation. The effect of *Asxl2* deficiency on bulk H3K27me2/3 levels suggests that in the adult heart, most PRC2 targets require ASXL2. In contrast, PHF1 may be required for the regulation of just a small number of targets.

Finally, although a GAL4-PHF1 fusion protein is able to recruit PRC2 to transgenic UAS sites, EZH2 enrichment at target chromatin is independent of PHF1 [38]. In comparison, ASXL2 is more critically required for PRC2-chromatin association at its target loci. This suggests that the two proteins use different mechanisms for promoting H3K27 trimethylation. For example, for PRC2 to efficiently convert H3K27me2 to H3K27me3 on chromatin substrate, there might be two prerequisites: stable chromatin association, followed by stimulation of enzymatic activity by a co-factor that can be independently recruited to target chromatin. We propose that ASXL2 regulates the first step, while PHF1 acts as a PRC2 co-factor.

### A potential link between histone H2A deubiquitination and H3K27 trimethylation?

Asx and ASXL proteins are core components of the PR–DUB complex, which specifically removes ubiquitin from histone H2A that is mono-ubiquitinated at lysine 119 [14]. The discovery that ASXL is required for PRC2 binding at target genes raises the question of whether PR–DUB deubiquitinase activity is involved in the regulation of PRC2 binding. In the mouse heart, ASXL2 is required for the homeostasis of both H3K27me3 and uH2A: the loss of *Asxl2* resulted in a decrease in the level of bulk H3K27me3 [19] as well as an increase in the level of bulk uH2A (Figure 9B). It remains to be answered whether there is any causative link between the changes in these two histone marks. On the other hand, in the hematopoietic cell lines studied by Abdel-Wahab et al., the loss of *ASXL1* disrupted PRC2 and H3K27me3 enrichment at the *HOXA* gene cluster without disrupting the level of uH2A [40]. Furthermore, knocking down BAP1 in the hematopoietic cell lines inactivated PR–DUB but did not reproduce the de-repression of *HOXA* genes as observed in *ASXL1*-deficient cells [40]. This seems to suggest that PR–DUB and PRC2 act independently of each other at the *HOXA* cluster, and that the loss of PRC2 recruitment in *ASXL1*-deficient cells did not result from inactivation of PR–DUB. A comprehensive study of more gene loci is needed to answer whether there is a functional relationship between histone H2A deubiquitination and H3K27 trimethylation. It is also possible that this relationship is different in heart tissue and in blood cells.

### Potential PR-DUB-independent mechanisms to regulate PRC2 binding

ASXL1/2 are large proteins that interact with multiple proteins other than BAP1 [43–45]. Interaction with histone and DNA has also been proposed [46]. These interactions could translate into PR–DUB-independent regulation of PRC2 binding. In mammalian cells, ASXL1 and ASXL2 co-purify with the YY1 protein in a >1 MD, multi-subunit complex [43]. The Drosophila homolog of YY1, Pleiohomeotic (Pho), is a sequence-specific DNA-binding protein that mediates the recruitment of other PcG proteins, including PRC2, to a subset of target chromatin sites [47–49]. When expressed in Drosophila, YY1 can rescue the homeotic phenotypes in homozygous *Pho* mutants, suggesting a high degree of functional conservation [50]. In mouse embryos, YY1 was found to co-localize with other PcG proteins and with H3K27me3 to upstream regulatory regions of *Hoxc8* and *Hoxa5* [51]. Through its interaction with YY1, ASXL2 could potentially regulate YY1’s ability to bind regulatory elements or other PcG proteins, thereby regulating PRC2 binding.

Asx and all ASXL proteins contain a highly conserved plant homeo domain (PHD) at the C-terminus [52]. The PHD finger is not involved in interaction with Calypso/Bap1 [14], yet is required for repression of *Ubx* in the wing primordia [53]. PHD fingers are found in many chromatin proteins and can mediate interactions with histones or non-histone protein partners [54]. For example, the PHD finger of Pcl is involved in binding to E(z) [41], and that of BPTF binds H3K4me3 [55,56]. If the PHD finger of ASXL2 interacts with PRC2 component(s) and/or with the nucleosome, it could directly contribute to PRC2 binding and/or to stabilizing PRC2 association with target chromatin.

A recent computational modeling study of ASXL proteins identified an N-terminal winged helix-turn-helix (wHTH) domain that is predicted to bind DNA [46]. wHTH domains are found in a number of eukaryotic and prokaryotic proteins that are known to bind DNA, including certain restriction endonucleases, DNA glycosylases, and the RNA polymerase delta subunit of Gram-positive bacteria. A wHTH-DNA interaction may increase the affinity of ASXL2/PRC2 to chromatin.

### Functional divergence between Asx and ASXL

The level of bulk H3K27me3 was dependent on ASXL1/2 in mammalian cells but was unaffected in Drosophila embryos carrying a homozygous null mutation of *Asx* [14]. Moreover, RNAi knock-down of *trx* severely disrupted binding of Asx, but not of PRC2, to polytene chromosomes [57], suggesting that PRC2 does not require Asx for chromatin association in Drosophila. What could account for this apparent discrepancy between the functional requirements for Drosophila Asx and for mouse ASXL1/2?

While the mechanism that regulates PRC2 binding is far from well understood, differences between mammals and Drosophila have been observed [4]. ASXL proteins may have evolved new functions, not possessed by Asx, to meet the specific needs of PRC2 regulation in mammals. Two lines of evidence are consistent with the scenario of functional divergence. First, although Asx family proteins range in size from 1370 to 2204-aa, homology between Asx and ASXL is largely restricted to the 32-aa PHD domain and the 120-aa ASXH domain [52]. Secondly, while PRC2 and ASXL1/2 co-IP in human cells [40] and mouse tissue (Figure 8E), Asx did not co-purify with Drosophila PRC2 in cultured cells [14].

Alternatively, the role of Asx/ASXL in PRC2 binding to chromatin may be dependent on the chromatin loci and/or on the cell type. For example, we showed that not all PcG targets require *Asxl2* for H3K27 trimethylation in the heart (Figure 5E, Figure 6E, Figure S4). The ratio of Asx/ASXL-dependent targets versus independent targets in a given tissue at a given developmental time may determine whether there is a detectable change in the level of bulk H3K27me3 in the mutant.

## Materials and Methods

### Animals

All mice used in this study were in C57BL/6J x 129Sv F1 background. This study was carried out in strict accordance with the recommendations in the Guide for the Care and Use of Laboratory Animals of the National Institutes of Health. The animal protocols were approved by the Animal Care Committee (ACC) at the University of Illinois at Chicago.

### Real-time RT-PCR

Total RNA was extracted from 1-month-old hearts and real-time RT-PCRs were performed using the SuperScript™ III Platinum SYBR Green One-Step qRT-PCR kit (Invitrogen).

Gene expression levels were normalized against that of 18S rRNA or *β-Actin* in the same sample. Primer sequences are provided in the Supplementary Material.

### Biochemical fractionation

Whole hearts were cut into pieces and homogenized in Buffer A (10 mM HEPES, pH 7.9, 10 mM KCl, 1.5 mM MgCl2, 0.34 M sucrose, 10% glycerol, 1 mM DTT, and protease inhibitors) using a Tissue Master homogenizer (OMNI International). Biochemical fractionation was performed as previously described [20].

### Chromatin immunoprecipitation (ChIP)

Nuclei were harvested from 1-month-old hearts that had been fixed in formaldehyde and homogenized. Chromatin was sheared by sonication andimmunoprecipitated with KC17 anti-ASXL2 antibody [21], anti-EZH2 antibody (Millipore), anti-SUZ12 antibody (Santa Cruz Biotechnology), anti-H3K27me3 (Abcam) or rabbit IgG (Invitrogen). ChIP-ed DNA was analyzed by PCR or real-time PCR. Primer sequences are provided in the Supplementary Material.

### Immunoprecipitation

Nuclear pellet was prepared from homogenized whole hearts and extracted in high salt buffer (50mM HEPES, 300mM NaCl, 10mM NaF, 1mM EDTA, 1% Triton-X, 1mM Na _3_VO_4_). Immunoprecipitations were performed using antibodies against proteins of interest and Dynabeads Protein G (Invitrogen). After washing, beads were boiled in Laemmli Buffer and IPed proteins were analyzed by Western blots.

## Supporting Information

Table S1 **Genes that are de-repressed or repressed by at least two-fold in Asxl2^-/-^ hearts, as determined by microarray analysis.**
(DOC)

Figure S1 **Epigenetic profiles at *Sfrp2*, *Acta1* and *Grk5* loci in ES cells.** The Broad Institute ChIP-seq database (http://www.broadinstitute.org/scientific-community/science/programs/epigenomics/chip-seqdata) was queried for the enrichment of H3K27me3, SUZ12, and EZH2 at the loci of interest. For each gene, only the genomic region around the TSS is shown. The scale bar for each panel is shown at the bottom of the panel. Arrow points to the direction of transcription. The y axis is the relative level of enrichment. (A–C) Representative epigenetic profiles for three types of genes in ES cells: those that are repressed by PcG activity, those that are constitutively expressed and not regulated by PcG activity, and those that are repressed via PcG-independent mechanism. (A) The chromatin region near the TSS of *Hoxa3*, a classical PcG target gene, displays high levels of enrichment of H3K27me3, SUZ12 and EZH2. (B) The profile for *Polr2d*, a housekeeping gene that encodes an RNA polymerase II subunit, shows no enrichment of H3K27me3, SUZ12 or EZH2. (C) H3K27me3 and PRC2 components are not enriched near the TSS of *Cp*, a gene that is repressed in ES cells. (D–F) The epigenetic profiles around the TSS of *Sfrp2*, *Acta1* and *Grk5* resemble that for *Hoxa3*.
(TIF)

Figure S2 **ASXL2 is not enriched at the *S100a10* locus.** *S100a10* encodes a calcium binding protein and is highly expressed in both wild-type and *Asxl2* ^*-/-*^ hearts. Shown are anti-ASXL2 ChIP-PCR results for six chromatin sites (a1-a6) within -5kb to +5kb of *S100a10* TSS. Mock ChIP was performed with normal rabbit IgG. Input: PCR assay of 1:100 diluted total input chromatin.
(TIF)

Figure S3 **ChIP-qPCR analysis of H3K27me3 enrichment at β*-MHC* (A–B), *Grk5* (C–D), *Sfrp2* (E–F) and *Acta1* (G–H) loci, shown as percentages of total input.** (A, C, E, G) H3K27me3 ChIP. (B, D, F, H) Mock IgG ChIP. Each column represents the mean value of data from three independent samples. *p<0.05; **p<0.01; Error bar: standard deviation.
(TIF)

Figure S4 **ChIP-qPCR analysis of H3K27me3 enrichment at the *Hoxb5* locus, shown as percentages of total input.** (A) Alignment of mouse, rat and human genomic sequences from -3kb to +3kb of *Hoxb5*. H1 and H2 are two highly conserved regions that were selected for ChIP-qPCR analysis. (B) H3K27me3 ChIP. (C) Mock IgG ChIP. Each column represents the mean value of data from three independent samples. Error bar: standard deviation.
(TIF)

Figure S5 **Comparison of EZH2 protein level in wild-type and *Asxl2*^*-/-*^ hearts**. Serial dilutions of heart extracts were subjected to SDS-PAGE and then probed with anti-EZH2 antibody. Western blot of TBP was used as a loading control.
(TIF)

Figure S6 **ChIP-qPCR analysis of EZH2 enrichment at β*-MHC* (A–B), *Grk5* (C–D), *Sfrp2* (E–F) and *Acta1* (G–H) loci, shown as percentages of total input.** (A, C, E, G) EZH2 ChIP. (B, D, F, H) Mock IgG ChIP. Each column represents the mean value of data from three independent samples. *p<0.05; **p<0.01; Error bar: standard deviation.
(TIF)

Figure S7 **Expression of *Asxl* genes in the adult mouse heart.** The mRNA levels of *Asxl1*, *Asxl2*, and *Asxl3* in wild-type and *Asxl2* ^*-/-*^ hearts were analyzed by real-time RT-PCR. Each column shown is the mean value of data generated from three independent samples. *p<0.05; Error bar: standard deviation.
(TIF)

Methods S1 **Supporting Methods.**
(DOC)
