# Supplementary material for: Additional Sex Combs-Like 2 Is Required for Polycomb Repressive Complex 2 Binding at Select Targets
Source: PLoS One. 2013 Sep 9;8(9):e73983. doi: 10.1371/journal.pone.0073983 (PMC3767597; doi:10.1371/journal.pone.0073983)
Supplement: Table S1 — Genes that are de-repressed or repressed by at least two-fold in Asxl2-/- hearts, as determined by microarray analysis. (DOC) [file pone.0073983.s001.doc]

SUPPLEMENTARY TABLE 1. Genes that are de-repressed or repressed by at least two-fold in Asx/2-1-  hearts, as determined by microarray analysis_

334 Genes derepressed for >= 2 fold in mutant hearts	
GeneID	gene	
71911	3-hydroxybutyrate dehydrogenase (heart, mitochondrial)	
22628	3-monooxygenase/tryptophan 5-monooxygenase activation protein , gamma polypeptide	
11431	acid phosphatase 1, soluble	
11459	actin, alpha  1, skeletal muscle	
71985	Acyl-Coenzyme A dehydrogenase family, member 10	
77794	ADAMTS-like  2	
269959	ADAMTS-like  3	
110532	adenosine deaminase , RNA-specific , Bl	
211673	ADP-ribosylation factor guanine nucleotide-exchange factor l(brefeldin A-inhibited)	
11569	AE binding protein 2	
22589	alpha thalassemia /mental retardation syndrome X-linked homolog (human)	
235633	ALS2 C-terminal like	
11820	Amyloid beta (A4) precursor protein	
70827	amyotrophic lateral sclerosis 2 (juvenile) chromosome region , candidate 3 (human)	
74018	Amyotrophic lateral sclerosis 2 (juvenile) homolog (human)	
57875	angiopoietin-like 4	
68743	anillin, actin binding protein (scraps homolog, Drosophila)	
17345	antigen identified by monoclonal antibody Ki 67	
11622	aryl-hydrocarbon receptor	
66929	ASFl anti-silencing function 1 homolog B (S. cerevisiae)	
23808	ash2 (absent, small, or homeotic)-like (Drosophila)	
104112	ATP citrate lyase	
70472	ATPase family, AAA domain containing 2	
235574	ATPase , Ca++-sequestering	
320940	Atpase, class VI, type 11C	
76184	ATP-binding cassette, sub-family A (ABCl), member 6	
13876	avian erythroblastosis virus E-26 (v-ets) oncogene related	
17940	baculoviral IAP repeat-containing la	
17948	baculoviral IAP repeat-containing lb	
11798	baculoviral IAP repeat-containing 4	
11799	baculoviral IAP repeat-containing 5	
12020	bagpipe homeobox gene 1 homolog (Drosophila)	
94043	Beta-amyloid binding protein precursor	
12064	brain derived neurotrophic factor	
12372	calsequestrin  1	
12349	carbonic anhydrase 2	
12319	carbonic anhydrase 8	
55987	carboxypeptidase X 2 (Ml4 family)	
12894	camitine palmitoyltransferase  la, liver	
13030	Cathepsin B	
235505	CD 109 antigen	
12484	CD24a antigen	
68916	CDK5 regulatory subunit associated protein 1-like 1	


105278	cell cycle related kinase	
12534	cell division cycle 2 homolog A (S. pombe)	
107995	cell division cycle 20 homolog (S. cerevisiae)	
52276	cell division cycle associated 8	
216991	centaurin , alpha 2	
12585	cerebellar  degeneration-related   2	
12870	ceruloplasmin	
12772	chemokine (C-C motif) receptor 2	
13051	chemokine (C-X3-C) receptor  I	
57266	chemokine (C-X-C motif) ligand 14	
57349	chemokine (C-X-C motif) ligand 7	
224796	chloride intracellular channel 5	
13004	chondroitin sulfate proteoglycan 3	
12667	chordin	
107932	chromodomain helicase DNA binding protein 4	
67337	cleavage stimulation factor, 3' pre-RNA , subunit I	
66983	coiled-coil domain containing 16	
235415	complexin  3	
194231	connector enhancer of kinase suppressor of Ras 1	
69274	CTD (carboxy-terminal domain, RNA polymerase II, polypeptide A) small phosphatase-like	
23845	C-type lectin domain family 5, member a	
74322	CXXC finger 1 (PHD domain)	
12428	cyclin A2	
12576	cyclin-dependent kinase inhibitor lB (P27)	
50766	Cysteine-rich motor neuron 1	
230459	cytochrome P450, family 2, subfamily j, polypeptide 13	
74519	Cytochrome P450, family 2, subfamily j , polypeptide 9	
70101	cytochrome P450, family 4, subfamily f, polypeptide 16	
12877	cytoplasmic  polyadenylation  element binding  protein  I	
80986	cytoskeleton associated protein 2	
212880	DEAD (Asp-Glu-Ala-Asp) box polypeptide 46	
13388	delta-like  I  (Drosophila)	
14357	Deltex 1 homolog (Drosophila)	
13356	DiGeorge syndrome critical region gene 2	
66369	dihydrouridine synthase 2-like (SMMl, S. cerevisiae)	
13482	dipeptidylpeptidase 4	
80915	dual specificity phosphatase 12	
13429	Dynamin   I	
13591	Early B-cell factor 1	
93685	ectonucleoside triphosphate diphosphohydrolase 7	
230316	EGF-like-domain , multiple 5	
13639	ephrin A4	
13642	ephrin B2	
77781	EPM2A (laforin) interacting protein  I	
71889	epsin 3	
192193	ER degradation enhancer, mannosidase alpha-like I	
269587	erythrocyte protein band 4.1	


170812	erythroid associated factor	
26380	estrogen related receptor , beta	
239528	eukaryotic translation initiation factor 2C, 2	
116701	fibroblast growth factor receptor-like I	
20378	frizzled-related protein	
56095	FtsJ homolog 3 (E. coli)	
73068	fucosyltransferase   11	
233079	G protein-coupled receptor 43	
14773	G protein-coupled receptor kinase 5	
118454	gap junction membrane channel protein alpha 12	
233863	genera l transcription  factor III C  I	
23885	germ cell-less homolog (Drosophila)	
384009	GLI pathogenesis-related 2	
14585	glial cell line derived neurotrophic factor family receptor alpha I	
14809	glutamate receptor , ionotropic , kainate 5 (gamma 2)	
76282	glutamic pyruvic transaminase 1, soluble	
14594	glycoprotein galactosyltransferase alpha I , 3	
94221	golgi associated PDZ and coiled-coil motif containing	
13197	growth arrest and DNA-damage-inducible 45 alpha	
210710	growth factor receptor bound protein 2-associated protein 3	
14544	guanine deaminase	
14695	guanine nucleotide binding protein , beta 3	
70676	GULP, engulfment adaptor PTB domain containing I	
56422	Hbs I -like (S. cerevisiae)	
233490	HCF-binding transcription factor Zhangfei	
15482	heat shock protein I-like	
23908	Heparan sulfate 2-0-sulfotransferase  I	
171285	hepatitis A virus cellular receptor 2	
50926	heterogeneous nuclear ribonucleoprotein D-like	
433785 Ill 433788	high mobility group box 2 Ill similar to high mobility group protein B2	
15466	histamine receptor H 2	
15115	Histidyl-tRNA synthetase	
14950	Histocompatibility 13	
319165	Histone I, H2ad Ill CDNA clone MGC: 103288 IMAGE:5150365, complete eds	
79221	histone deacetylase 9	
27281	HRAS-like suppressor	
330790	hyaluronan and proteoglycan link protein 4	
15366	hyaluronan mediated motility receptor (RHAMM)	
15586 Ill 56441	hyaluronidase I Ill N-acetyltransferase 6	
15483	hydroxysteroid  I I-beta dehydrogenase  I	
16324	inhibin beta-B	
16150	inhibitor ofkappaB kinase beta	
16319	inner centromere protein	
233011	inositol 1,4,5-trisphosphate 3-kinase C	
223272	integrin, beta-like  I	
57444	interferon-stimulated protein	
16477	Jun-B oncogene	


19348	kinesin family member 20A	
16570	kinesin family member 3C	
21847	Kruppel-like factor I 0	
67557	La ribonucleoprotein domain family , member 6	
16833	lactate dehydrogenase 3, C chain , sperm specific	
71835	LanC (bacterial !antibiotic synthetase component C)-like 2	
93730	Leucine zipper transcription factor-like I	
329252	leucine-rich repeat-containing G protein-coupled receptor 6	
74201	leucine-rich repeats and IQ motif containing 2	
16840	leukocyte cell derived chemotaxin  I	
16881	ligase I, DNA , ATP-dependent	
16826	LIM domain binding 2	
69605	limb and neural patterns	
64899	lipin 3	
100702	macrophage activation 2 like	
54484	makorin, ring finger protein , I	
17389	matrix metalloproteinase 16	
104362	meiosis expressed gene  I	
17765	Metal response element binding transcription factor 2	
17768	methylenetetrahydrofolate dehydrogenase (NAD+ dependent) , methenyltetrahydrofolate cyclohydrolase	
216760	microfibrillar-associated protein 3	
171580	microtubule associated monoxygenase, calponin and LIM domain containing I	
225164	mindbomb homolog I (Drosophila)	
17215	minichromosome maintenance deficient 3 (S. cerevisiae)	
17218	minichromosome maintenance deficient 5, cell division cycle 46 (S. cerevisiae)	
17219	minichromosome maintenance deficient 6 (MISS homolog, S. pombe) (S. cerevisiae)	
26400	mitogen activated protein kinase kinase 7	
17344	Msx-interacting-zinc finger	
76626	Musashi homolog 2 (Drosophila)	
27418	Muskelin I, intracellular mediator containing kelch motifs	
17153	myelin and lymphocyte protein , T-cell differentiation protein	
233199	myosin binding protein C, fast-type	
74376	myosin XVIIIb	
18104	NAD(P)H  dehydrogenase,  quinone  I	
234258	nei like 3 (E. coli)	
235627	neurobeachin-like  2	
74513	neuropilin (NRP) and tolloid (TLL)-like 2	
74455	NOL1/NOP2 / Sun domain family 6	
108907	nucleolar and spindle associated protein I	
67528	nudix (nucleoside diphosphate linked moiety X)-type motif7	
68767	open reading frame 19	
231805	paired immunoglobin-like type 2 receptor alpha	
18933	paired related homeobox  1	
19228	parathyroid  hormone receptor  I	
18771	Pbx/knotted  1 homeobox	
56376	PDZ and LIM domain 5	
170761	PDZ domain containing 2	


237504	peptidylglycine alpha-amidating monooxygenase COOR-terminal interactor	
75725	PHD finger protein 14	
74769	phosphatidylinositol 3-kinase, catalytic, beta polypeptide	
18578	phosphodiesterase 4B, cAMP specific	
107272	phosphoserine aminotransferase  l	
18792	plasminogen activator, urokinase	
71785	platelet-derived growth factor, D polypeptide	
67448	plexin domain containing 2	
20873	polo-like kinase 4 (Drosophila)	
218832	polymerase (RNA) III (DNA directed) polypeptide A	
19285	polymerase I and transcript release factor	
16526	potassium channel , subfamily K, member 2	
16513	potassium inwardly-rectifying channel, subfamily J, member 10	
211480	potassium inwardly-rectifying channel, subfamily J, member 14	
70673	PR domain containing 16	
18514	Pre B-cell leukemia transcription factor 1	
12828	procollagen , type IV, alpha 3	
12837	procollagen, type VIII, alpha 1	
12817	procollagen , type XIII, alpha 1	
12818	procollagen , type XIV , alpha 1	
67505	prolactin like protein 0	
19200	praline-serine-threonine phosphatase-interacting protein 1	
19183	proteasome (prosome, macropain) 26S subunit, ATPase 3, interacting protein	
80708	protein kinase C and casein kinase substrate in neurons 3	
19106	protein kinase, interferon-inducible double stranded RNA dependent	
69847	protein kinase, lysine deficient 4	
19055	Protein phosphatase 3, catalytic subunit, alpha isoform	
233406	protein regulator of cytokinesis 1	
19263	protein tyrosine phosphatase , receptor type , B	
19268	protein tyrosine phosphatase , receptor type , F	
53601	protocadherin 12	
14083	PTK2 protein tyrosine kinase 2	
18546	Purkinje cell protein 4	
26934	Rae GTPase-activating protein I	
19362	RAD5 l associated protein 1	
19361	RADS! homolog (S. cerevisiae)	
58185	radical S-adenosyl methionine domain containing 2	
192786	Rap guanine nucleotide exchange factor (GEF) 6	
51869	Rap 1 interacting factor 1 homolog (yeast)	
19883	RAR-related orphan receptor alpha	
192656	receptor (TNFRSF)-interacting serine-threonine kinase 2	
19724	regulatory factor X , I (influences HLA class II expression)	
214742	REST corepressor 3	
65079	reticulon 4 receptor	
19889	retinitis pigmentosa 2 homolog (human)	
328365	Retinoic acid induced 17	
105014	retinal dehydrogenase 14 (all-trans and 9-cis)	


103142	retinol dehydrogenase 9	
75415	Rho GTPase activating protein 12	
19819	ribonuclease HI	
382985	ribonucleotide reductase M2 B (TP53 inducible)	
20088	ribosomal protein S24	
110651	ribosomal protein S6 kinase polypeptide 3	
30054	ring finger protein  I 7	
19881	rod outer segment membrane protein  1	
230257	RODI regulator of differentiation I (S. pombe)	
229675	rosbin , round spermatid basic protein I	
20202	SIOO calcium binding protein A9 (calgranulin B)	
83997	sarcolemma associated protein	
20319	secreted frizzled-related sequence protein 2	
22287	secretoglobin, family IA , member 1 (uteroglobin)	
56747	seizure related 6 homolog (mouse)-like	
233878	seizure related 6 homolog (mouse)-like 2	
20347	sema domain , immunoglobulin domain (lg), short basic domain , secreted , (semaphorin) 3B	
20360	sema domain, transmembrane domain (TM), and cytoplasmic domain, (semaphorin) 6C	
214968	sema domain , transmembrane domain (TM), and cytoplasmic domain , (semaphorin) 6D	
18787	serine (or cysteine) proteinase inhibitor , clade E, member 1	
20817	serine/arginine-rich protein specific kinase 2	
66244	serologically defined colon cancer antigen 1	
20437 /// 20438	seven in absentia 1A /// seven in absentia lB	
269016	SH3 domain containing ring finger 2	
59009	SH3 multiple domains 2	
20419	She SH2-domain binding protein 1	
20612	sialoadhesin	
272713	Similar to development- and differentiation-enhancing factor 2; PYK2 C terminus-associated protein	
546041	similar to p47 protein isoform a	
384254	similar to ribosomal protein L18a	
20292	small chemokine (C-C motif) ligand 11	
30927	snail homolog 3 (Drosophila)	
399548	sodium channel , type IV, beta	
65221	solute carrier family 15, member 3	
105355	solute carrier family 17 (sodium phosphate) , member 3	
20515	solute carrier family 20, member I	
67712	solute carrier family 25, member 37	
215085	solute carrier family 35, member FI	
106957	solute carrier family 39 (metal ion transporter), member 6	
20533	solute carrier family 4 (anion exchanger) , member I	
20537	solute carrier family 5 (sodium/glucose cotransporter), member 1	
20658	Son cell proliferation protein	
20662	Son of sevenless homo log 1 (Drosophila)	
20411	Sorbin and SH3 domain containing I	
109552	sorein	
20660	sortilin-related receptor , LDLR class A repeats-containing	
432572	spectrin domain with coiled-coils 1	


66234	Sterol-C4-methyl oxidase-like	
67902	sulfatase modifying factor 2	
20975	synaptojanin  2	
54525	Synaptotagmin 7	
269397	synovial sarcoma translocation gene on chromosome 18-like l	
268996	synovial sarcoma translocation, Chromosome 18	
20617	synuclein , alpha	
230908	TAR DNA binding protein	
21923	tenascin C	
217449	tetratricopeptide repeat domain 15	
73666	THO complex 3	
58916	titin immunoglobulin domain protein (myotilin)	
22034	Tnf receptor-associated factor 6	
21899	toll-like receptor 6	
21973	topoisomerase (DNA) II alpha	
21974	topoisomerase (DNA) II beta	
21419	transcription factor AP-2 beta	
81004	transducin (beta)-like IX-linked receptor l	
67226	Transmembrane protein 19	
224090	transmembrane protein 44	
80890	tripartite motif protein 2	
217069	tripartite motif protein 25	
94089	tripartite motif protein 7	
22004	tropomyosin 2, beta	
21953	troponin I, skeletal , fast 2	
22035	tumor necrosis factor (ligand) superfamily, member 10	
79202	tumor necrosis factor receptor superfamily, member 22	
22222	ubiquitin protein ligase E3 component n-recognin l	
230484	ubiquitin specific protease 1	
319651	ubiquitin specific protease 37	
252870	ubiquitin  specific protease  7	
140499	ubiquitin-conjugating enzyme E2, J2 homolog (yeast)	
68612	ubiquitin-conjugating enzyme E2C	
56791	ubiquitin-conjugating enzyme E2L 6	
18140	ubiquitin-like , containing PHD and RING finger domains , 1	
22236 Ill 39443012 13141516 Ill 94284	UDP glucuronosyltransferase l family, polypeptide A2 Ill A6A Ill AlO Ill A7C Ill A5 Ill A9 Ill A6B Ill Al	
320011	UDP-glucose ceramide glucosyltransfera se-like l	
22229	uncoupling protein 3 (mitochondrial, proton carrier)	
171530	urocortin  2	
22310 Ill 22311	vomeronasa l 2, receptor , 4 Ill vomeronasa l 2, receptor, 5	
73674	WD repeat domain 75	
22376	Wiskott-Aldrich syndrome homolog (human)	
229055	zinc finger and BTB domain containing l 0	
268294	Zinc finger and BTB domain containing 24	
16969	zinc finger and BTB domain containing 7a	
106205	zinc finger CCCH type containing 7	
22697	zinc finger proliferation l	


22666	zinc finger protein 161	
193452	zinc finger protein 184 (Kruppel-like)	
101095	zinc finger protein 282	
68910	zinc finger protein 467	
218820	zinc finger protein 503	
22775	zinc finger protein interacting with K protein 1	
22764	zinc finger protein X-linked	
224454	zinc finger, DHHC domain containing 14	
75965	zinc finger, DHHC domain containing 20	


419 Genes repressed for>= 2 fold in mutant hearts	
GenelD	gene	
246729	2'-5' oligoadenylate synthetase lH	
246728	2'-5' oligoadenylate synthetase 2	
18640	6-phosphofructo-2-kinase/fructose-2,6-biphosphatase   2	
13522	a disintegrin and metalloprotease domain 28	
11475	actin, alpha 2, smooth muscle, aorta	
11474	actinin alpha 3	
216739	acyl-CoA synthetase long-chain family member 6	
75302	additional sex combs like 2 (Drosophila)	
11450	adiponectin, ClQ and collagen domain containing	
11537	adipsin	
218639	ADP-ribosylation factor related protein 2	
11875	ADP-ribosyltransferase   5	
14266	AF4/FMR2 family, member 2	
93736	AF4/FMR2 family, member 4	
211064	alkB, alkylation repair homolog (E.coli)	
232345	alpha-2-macroglobulin	
23923	aminoadipate  aminotransferase	
72823	Amyotrophic lateral sclerosis 2 (juvenile) chromosome region, candidate 19	
11600	angiopoietin 1	
70008	angiotensin I converting enzyme (peptidyl-dipeptidase A) 2	
70797	Ankyrin repeat and IBR domain containing 1	
208117	anterior pharynx defective lb homolog (C. elegans)	
110542	anti-Mullerian hormone type 2 receptor	
11800	apoptosis inhibitor 5	
11829	aquaporin 4	
11831	aquaporin 6	
70882	armadillo repeat containing 3	
11899	astrotactin 1	
72174	Ataxin 7-like 4	
11946	ATP synthase, H+ transporting, mitochondrial Fl complex, alpha subunit, isoform 1	
50771	ATPas, class II, type 9B	
70472	ATPase family, AAA domain containing 2	
74772	ATPase type 13A2	
11975	ATPase, H+ transporting, lysosomal VO subunit a isoform 1	
11944	ATPase, H+/K+ transporting, alpha polypeptide	
11304	ATP-binding cassette, sub-family A (ABCl), member 4	
26874	ATP-binding cassette, sub-family D (ALO), member 2	
27409	ATP-binding cassette, sub-family G (WHITE), member 5	
66898	BAil-associated protein 2-like 1	
14025	B-cell CLL/lymphoma llA (zinc finger protein)	
12175	BCL2/adenovirus ElB 19kDa-interacting protein 1, NIP2	
70026	benzodiazapine receptor, peripheral-like 1	
12234	Beta-transducin repeat containing protein	
76895	bicaudal D homolog 2 (Drosophila)	
75770	BR serine/threonine  kinase 2	


109880	Braf transforming gene	
108100	brain-specific angiogenesis inhibitor 1-associated protein 2	
76960	breast carcinoma amplified sequence 1	
192197	breast carcinoma amplified sequence 3	
76809	Bri3 binding protein	
22385	bromodomain adjacent to zinc finger domain, lB	
12300	calcium channel, voltage-dependent, gamma subunit 2	
12288	Calcium channel, voltage-dependent , L type, alpha lC subunit	
227541	Calcium/calmodulin-dependent  protein kinase ID	
12374	calcium-sensing receptor	
442829	calicin	
231991	cAMP responsive element binding protein 5	
12350	carbonic anhydrase 3	
104158	carboxylesterase  3	
17079	CD180 antigen	
12478	CD19 antigen	
171486	Cd99 antigen-like 2	
229776	CDC14 cell division cycle 14 homolog A (5. cerevisiae)	
14311	cell death-inducing DFFA-like effector c	
212285	centaurin, delta 1	
108000	centromere  autoantigen   F	
12622	cerberus 1homolog (Xenopus laevis)	
12405	cerebellin 2 precursor protein	
105513	CHMP family,member 7	
75677	claudin 22	
12739	claudin 3	
66098	Coiled-coil-helix-coiled-coil-helix  domain containing 6	
320924	Collagen and calcium binding EGF domains 1	
14219	connective tissue growth factor	
21367	contactin 2	
18488	contactin 3	
12808	cordon-bleu	
224912	crumbs homolog 3 (Drosophila)	
12965 /// 12966	crystallin, gamma B /// crystallin, gamma C	
51811	C-type lectin domain family 4,member f	
74100	cyclic AMP-regulated phosphoprotein ,21	
51813	Cyclin C	
94219	cyclin M2	
13008	cysteine and glycine-rich protein 2	
12583	cysteine dioxygenase 1,cytosolic	
13075	cytochrome P450, family 19, subfamily a, polypeptide 1	
13106	cytochrome P450, family 2, subfamily e, polypeptide 1	
231162	cytokine like 1	
229459	Dachsous 2 (Drosophila)	
66573	DAZ interacting protein 1	
74351	DEAD (Asp-Glu-Ala-Asp)  box polypeptide 23	
71412	dehydrogenase/reductase  member 2	


66705	deoxyribonuclease 1-like 2	
13367	diaphanous homolog 1(Drosophila)	
50781	dickkopf homolog 3 (Xenopus laevis)	
12305	Discoidin domain receptor family, member 1	
330938	DIX domain containing 1	
13434	DNA  methyltransferase  2	
73284	DNA-damage-inducible transcript 4-like	
13447	double C2, beta	
13175	double cortin and calcium/calmodulin-dependent protein kinase-like 1	
216164	downstream of Stkll	
18218	dual specificity phosphatase 8	
13656	early growth response 4	
67868	Elastase 3 B, pancreatic	
106389	ELL associated factor 2	
269344	elongation factor RNA polymerase II-like 3	
13796	empty spiracles homolog 1(Drosophila)	
13797	empty spiracles homolog 2 (Drosophila)	
107522	endothelin converting enzyme 2	
13839	Eph receptor AS	
18612	Ets variant gene 4 (ElA enhancer binding protein, ElAF)	
13664 /// 266459	eukaryotic translation initiation factor lA ///similar to Eukaryotic translation initiation factor lA (elF-lA) (elF-4C)	
230861	Eukaryotic translation initiation factor 4 gamma, 3	
13684	Eukaryotic translation initiation factor 4E	
14050	eyes absent 3 homolog (Drosophila)	
14104	fatty acid synthase	
314322	FBJ murine osteosarcoma viral oncogene homolog	
57443	F-box only protein 3	
14119	fibrillin 2	
14167	fibroblast growth factor 12	
14169	Fibroblast growth factor 14	
14264	fibromodulin	
15227	forkhead box Fla	
114142	forkhead box P2	
14221	four jointed box 1(Drosophila)	
14266	Fragile X mental retardation 2 homolog	
14352	Friend virus susceptibility 4	
57265	frizzled homolog 2 (Drosophila)	
67391	FUN14 domain containing 2	
213054	GA repeat binding protein, beta 2	
14403	Gamma-aminobutyric acid (GABA-A) receptor, subunit delta	
14611	gap junction membrane channel protein alpha 3	
14617	gap junction membrane channel protein alpha 9	
66790	GH regulated TBC protein 1	
14537	glucosaminyl (N-acetyl) transferase 1, core 2	
14538	glucosaminyl (N-acetyl) transferase 2, I-branching enzyme	
14810	glutamate receptor, ionotropic, NMDAl (zeta 1)	
14583	glutamine fructose-6-phosphate transaminase 1	


68312	glutathione 5-transferase, mu 7	
227960	grancalcin	
78926	growth arrest-specific 2 like 1	
14559 /// 93898	growth differentiation factor 1///longevity assurance homolog 1(5. cerevisiae)	
14582	growth factor independent lB	
14702	guanine  nucleotide  binding protein  (G protein), gamma  2 s ubunit	
14709	guanine nucleotide binding protein (G protein),gamma 8 subunit	
14955	H19 fetal liver mRNA	
15439	haptoglobin	
26386	heat shock transcription factor 4	
94253	HECT, C2 and WW domain containing E3 ubiquitin protein ligase 1	
59026	HECT, UBA and WWE domain containing 1	
84506	hepcidin antimicrobial peptide 1	
15388	Heterogeneous nuclear ribonucleoprotein L	
14950	histocompatibility  13	
14968	histocompatibility  2, cla ss II antigen E alpha	
68024	histone 1, H2bc	
15394	homeo box Al	
15424	homeo box CS	
75828	HORMA domain containing 2	
330723	HtrA serine peptidase 4	
16656	human immunodeficiency virus type I enhancer binding protein 3	
53323	huntingtin interacting protein 2	
77042	hyaluronoglucosaminidase 4	
18518	lmmunoglobulin (CD79A) binding protein 1	
16061	lmmunoglobulin heavy chain (J558 family)	
16017	immunoglobulin heavy chain 4 (serum lgGl)	
16071	lmmunoglobulin  kappa chain,constant  region	
16142	immunoglobulin lambda chain, variable 1	
209268	immunoglobulin superfamily, member 1	
54725	immunoglobulin superfamily, member 4A	
27993	IMP4,U3 small nucleolar ribonucleoprotein ,homolog (yeast)	
16438	Inositol 1,4,5-triphosphate receptor 1	
75426	insulin-like growth factor binding protein-like 1	
16398	integrin alpha 2	
270110	interferon regulatory factor 2 binding protein 2	
54450	interleukin 1family, member 5 (delta)	
50931	interleukin 27 receptor,alpha	
16190	interleukin 4 receptor,alpha	
75605	jumonji , AT rich interactive domain lB (Rbp2 like)	
57340	junctophilin  3	
63830	KCNQl overlapping transcript 1	
16673	keratin complex 1, acidic, gene 5	
110308	keratin complex 2, basic,gene 5	
16578	kinesin family member 9	
84035	kringle containing transmembrane protein 1	
237339	1(3)mbt-like 3 (Drosophila)	


79235	Lecithin-retinol  acyltransferase   (phosphatidylcholine-retinol-0-acyltransferase)	
70361	Lectin, mannose-binding, 1	
76612	leucine rich repeat containing 27	
216028	Leucine rich repeat transmembrane neuronal 3	
329252	leucine-rich repeat-containing G protein-coupled receptor 6	
16871	LIM homeobox protein 3	
16876	LIM homeobox protein 9	
68311	Ly6/Plaur domain containing 2	
23934	lymphocyte antigen 6 complex, locus H	
16842	Lymphoid enhancer binding factor 1	
15064	major histocompatibility complex, class I-related	
320772	MAM domain containing 1	
17174	mannan-binding lectin serine protease 1	
17160	mannosidase 2, alpha B2	
17184	Matrin 3	
17387	matrix metalloproteinase 14 (membrane-inserted)	
17122	Max dimerization protein 4	
56524	Membrane protein, palmitoylated 6 (MAGUK p55 subfamily member 6)	
170813	membrane-spanning 4-domains, subfamily A, member 3	
17752	metallothionein 4	
17150	microfibrillar-associated  protein 2	
71306	microfibrillar-associated protein 3-like	
328329	microtubule associated serine/threonine  kinase family member 4	
17318	midline 1	
66845	mitochondrial ribosomal protein L33	
60441	mitochondrial ribosomal protein L38	
23939	mitogen activated protein kinase 7	
60597	mitogen-activated  protein kinase 8 interacting protein 2	
83456	Moloney leukemia virus 10-like 1	
109731	monoamine oxidase  B	
67973	M-phase phosphoprotein 10 (U3 small nucleolar ribonucleoprotein)	
67014	myc induced nuclear antigen	
17536	Myeloid ecotropic viral integration site-related gene 1	
17260	Myocyte enhancer factor 2C	
17918	Myosin Va	
17901	myosin, light polypeptide 1	
17896	myosin, light polypeptide 4	
17898	myosin, light polypeptide 7, regulatory	
98932	myosin, light polypeptide 9, regulatory	
217214	N-acetylglutamate synthase	
230899	natriuretic peptide precursor type A	
53885	nephronophthisis  1Uuvenile)  homolog (human)	
57764	netrin 4	
80883	netrin Gl	
18011	neuralized-like homolog (Drosophila)	
235627	neurobeachin-like 2	
320840	neuronal growth regulator 1	


18211	neurotrophic tyrosine kinase, receptor,type 1	
18188	neurturin	
66866	NHL repeat containing 2	
18092	NK2 transcription factor related, locus 6 (Drosophila)	
18124	nuclear receptor subfamily 4, group A,member 3	
18226	Nucleoporin 62	
218121	0-acyltransferase (membrane bound) domain containing 1	
18300	oncoprotein induced transcript 1	
15379	one cut domain, family member 1	
56374	open reading frame 18	
80909	opposite strand transcription unit to Stag3	
330962	organic solute transporter beta	
70061	orphan short chain dehydrogenase/reductase	
105689	Pam,highwire,rpm 1	
93742	Par-3 (partitioning defective 3) homolog (C. elegans)	
53318	PDZ and LIM domain 3	
18599	peptidyl arginine deiminase, type I	
19132	Peripherin 1	
19679	phosphatidylinositol transfer  protein, membrane-associated  2	
18576	phosphodiesterase  3B,cGMP-inhibited	
29863	Phosphodiesterase  7B	
227120	phospholipase C-like 1	
110094	Phosphorylase kinase alpha 2	
56460	plakophilin 3	
68797	platelet-derived growth factor receptor-like	
215632	pleckstrin and Sec7 domain containing 4	
231999	pleckstrin homology domain containing, family A (phosphoinositide binding specific) member 8	
211945	pleckstrin homology domain containing, family H (with MyTH4 domain) member 1	
378460	PML-RAR alpha-regulated adaptor molecule 1	
319655	podocalyxin-like 2	
16519	potassium inwardly-rectifying channel, subfamily J, member 3	
16509	potassium voltage-gated channel, lsk-related subfamily, member 1	
16508	potassium voltage-gated channel, Shal-related family, member 2	
211468	Potassium voltage-gated channel, subfamily H (eag-related), member 8	
64243	Prader-Willi chromosome region 1homolog (human)	
237759	procollagen, type XXlll, alpha 1	
74229	progestin and adipoQ receptor family member VIII	
170952	proline rich membrane anchor 1	
65116	proline-rich Gia (G-carboxyglutamic acid) polypeptide 2	
19220	prostaglandin  F receptor	
67151	Proteasome (prosome, macropain) 265 subunit, non-ATPase, 9	
19088	protein kinase, cAMP dependent regulatory,type II beta	
19091	protein kinase, cGMP-dependent, type I	
320472	protein phosphatase lE (PP2C domain containing)	
68507	protein tyrosine phosphatase,receptor type, f polypeptide (PTPRF),interacting protein (liprin), alpha 4	
211712	protocadherin 9	
93886	protocadherin beta 15	


230596	PRP38 pre-mRNA processing factor 38 (yeast) domain containing A	
101631	PWWP domain containing 2	
18770	pyruvate kinase liver and red blood cell	
29809	RAB GTPase activating protein 1-like	
98710	RAB interacting factor	
227746	Rab9 effector protein with kelch motifs	
114714	Rad51 homolog c (S. cerevisiae)	
19364	RAD51-like 3 (S. cerevisiae)	
78255	Ral GEF with PH domain and SH3 binding motif 2	
19765	ralA binding protein 1	
320292	RasGEF domain family, member lB	
51801	receptor (calcitonin) activity modifying protein 1	
19715	reduced expression 2	
19716	reduced expression 3	
51791	regulator of G-protein signaling 14	
50779	regulator of G-protein signaling 6	
243923	regulator of G-protein s ignalling 9 binding protein	
328280	Regulator of sex-limitation candidate 24	
57262	resistin like alpha	
104001	reticulon 1	
19662	retinal binding protein 4, plasma	
171207	Rho GTPase activating protein 4	
19989	ribosomal protein L7	
68925	RNA polymerase II associated protein 1	
20191	Ryanodine receptor 2, cardiac	
66402	sarcolipin	
140740	SEC63-like (S. cerevisiae)	
20338	Sell (suppressor of lin-12} 1homolog (C. elegans)	
20350	sema domain, immunoglobulin domain (lg), short basic domain,secreted, (semaphorin) 3 F	
20700111 20701/3/4 /// 544889	serine (or cysteine) proteinase inhibitor, clade A, member la/// lb/// ld ///le Ill similar to Serpinala protein	
12406	serine (or cysteine) proteinase inhibitor , clade H,member 1	
56726	SH3-binding domain glutamic acid-rich protein like	
20404	SH3-domain GRB2-like 2	
237979	sidekick homolog 2 (chicken)	
107513	Signal sequence receptor, alpha	
432700	Similar to lg heavy chain V-111 region J606	
546611	similar to kelch-like 9	
435350	similar to NK13	
546020	similar to secreted gel-forming mucin	
20473	sine oculis-related homeobox 3 homolog (Drosophila)	
75345	SLAM family member 7	
20563	Slit homolog 2 (Drosophila)	
20365	small EDRK-rich factor 1	
20269	sodium channel,voltage-gated, type Ill, alpha	
20493	solute carrier family 10 (sodium/bile acid cotransporter family), member 1	
56643	solute carrier family 15 (oligopeptide transporter) ,member 1	
20503	solute carrier family 16 (monocarboxylic acid transporter s),member 7	


20519	solute carrier family 22 (organic cation transporter), member 3	
22232	solute carrier family 35 (UDP-galactose transporter), member 2	
58246	solute carrier family 35, member B4	
53945	solute carrier family 40 (iron-regulated transporter), member 1	
246787	solute carrier family 5 (sodium/glucose cotransporter), member 2	
230612	solute carrier family 5 (sodium/glucose cotransporter), member 9	
20541	solute carrier family 8 (sodium/calcium exchanger), member 1	
226999	solute carrier family 9 (sodium/hydrogen exchanger), member 2	
77031	solute carrier family 9 (sodium/hydrogen exchanger), member 8	
69024	Sorting nexin 15	
75469	spermatogenesis associated 19	
57815	spermatogenesis  associated  5	
27401	S-phase kinase-associated protein 2 (p45)	
56632	sphingosine kinase 2	
278240	spindlin-like	
114715	Sprouty protein with EVH-1domain1, related sequence	
20775	squalene epoxidase	
20669	SRY-box containing gene 14	
20680	SRY-box containing gene 7	
20447	ST6 (alpha-N-acetyl-neuraminyl-2,3-beta-galactosyl-1,3)-N-acetylgalactosaminide   alpha-2,6-sialyltransferase 3	
56018	START domain containing 10	
20249	stearoyl-Coenzyme A desaturase 1	
74480	sterile alpha motif domain containing 4	
78925	Steroid 5 alpha-reductase 1	
20655	Superoxide dismutase  1,soluble	
93762	SWl/SNF related, matrix associated, actin dependent regulator of chromatin, subfamily a, member 5	
214804	synapse defective 1, Rho GTPase, homolog 2 (C. elegans)	
64176	synaptic vesicle glycoprotein 2 b	
52440	Taxl (human T-cell leukemia virus type I) binding protein 1	
83993	T-box 19	
57246	T-box 20	
319939	tensin 3	
60600	Testis specific gene AS	
68498	tetraspanin 11	
223431	TGF-betal-induced  anti-apoptotic factor 2	
234723	thioredoxin- like 4B	
21834	thyroid hormone receptor beta	
76367	TP53 regulating kinase	
69014	TRAF2 and NCK interacting kinase	
21419	Transcription factor AP-2 beta	
252973	transcription factor CP2-like 3	
57259	transducer of ERBB2, 2	
21372	Transducin (beta)-like 1X-linked	
22059	transformation related protein 53	
21808	transforming growth factor, beta 2	
331046	transglutaminase 4 (prostate)	
67564	transmembrane  protein 35	


235135	transmembrane protein 45b	
227331	Trinucleotide repeat containing 15	
229644	tripartite motif-containing 45	
67525	tripartite motif-containing 62	
50876	tropomodulin 2	
319953	tubulin tyrosine ligase-like 1	
67534	tubulin tyrosine ligase-like family, member 4	
233276	Tubulin, gamma complex associated protein 5	
24099	tumor necrosis factor (ligand) superfamily, member 13b	
21939	tumor necrosis factor receptor superfamily, member 5	
22070	tumor protein, translationally-controlled 1	
13345	twist homolog 2 (Drosophila)	
234724	tyrosine aminotransferase	
83813	tyrosine kinase, non-receptor, 1	
212190	U8X domain containing 3	
93961	UDP-Gal:betaGlcNAc beta 1,3-galactosyltransferase, polypeptide 5	
14425	UDP-N-acetyl-alpha-D-galactosamine:polypeptide  N-acetylgalactosaminyltransferase 3	
22227	uncoupling protein 1(mitochondrial, proton carrier)	
22256	Uracil-DNA glycosylase	
103149	ureidopropionase,  beta	
338362	Uronyl-2-su lfotra nsferase	
22268	uroplakin 18	
100647	uroplakin 38	
22283	Usher syndrome 2A (autosomal recessive, mild) homolog (human)	
195434	UTP14, U3 small nucleolar ribonucleoprotein, homolog 8 (yeast)	
74199	vitrin	
83767	WASP family 1	
22408	wingless-related MMTV integration site 1	
22416	Wingless-related MMTV integration site 3A	
24117	Wnt inhibitory factor 1	
22402	WNTl inducible signaling pathway protein 1	
74254	XPA binding protein 1	
235320	Zinc finger and 8T8 domain containing 16	
75580	zinc finger and 8T8 domain containing 4	
16969	Zinc finger and 8T8 domain containing 7a	
56869	zinc finger protein 109	
57908	zinc finger protein 318	
328977	Zinc finger protein 532	
241494	zinc finger protein 533	
233887	zinc finger protein 553	
69234	zinc finger protein 688	
213436	zinc finger, CCHC domain containing 5	
433204111 66980	zinc finger, DHHC domain containing 6111 similar to Zinc finger  DHHC domain containing protein 6 (H4 homolog)	
53604	zona pellucida binding protein	
80292	ZXD family zinc finger C	
