# Supplementary figures and images for: Additional Sex Combs-Like 2 Is Required for Polycomb Repressive Complex 2 Binding at Select Targets
Source: PLoS One. 2013 Sep 9;8(9):e73983. doi: 10.1371/journal.pone.0073983 (PMC3767597; doi:10.1371/journal.pone.0073983)

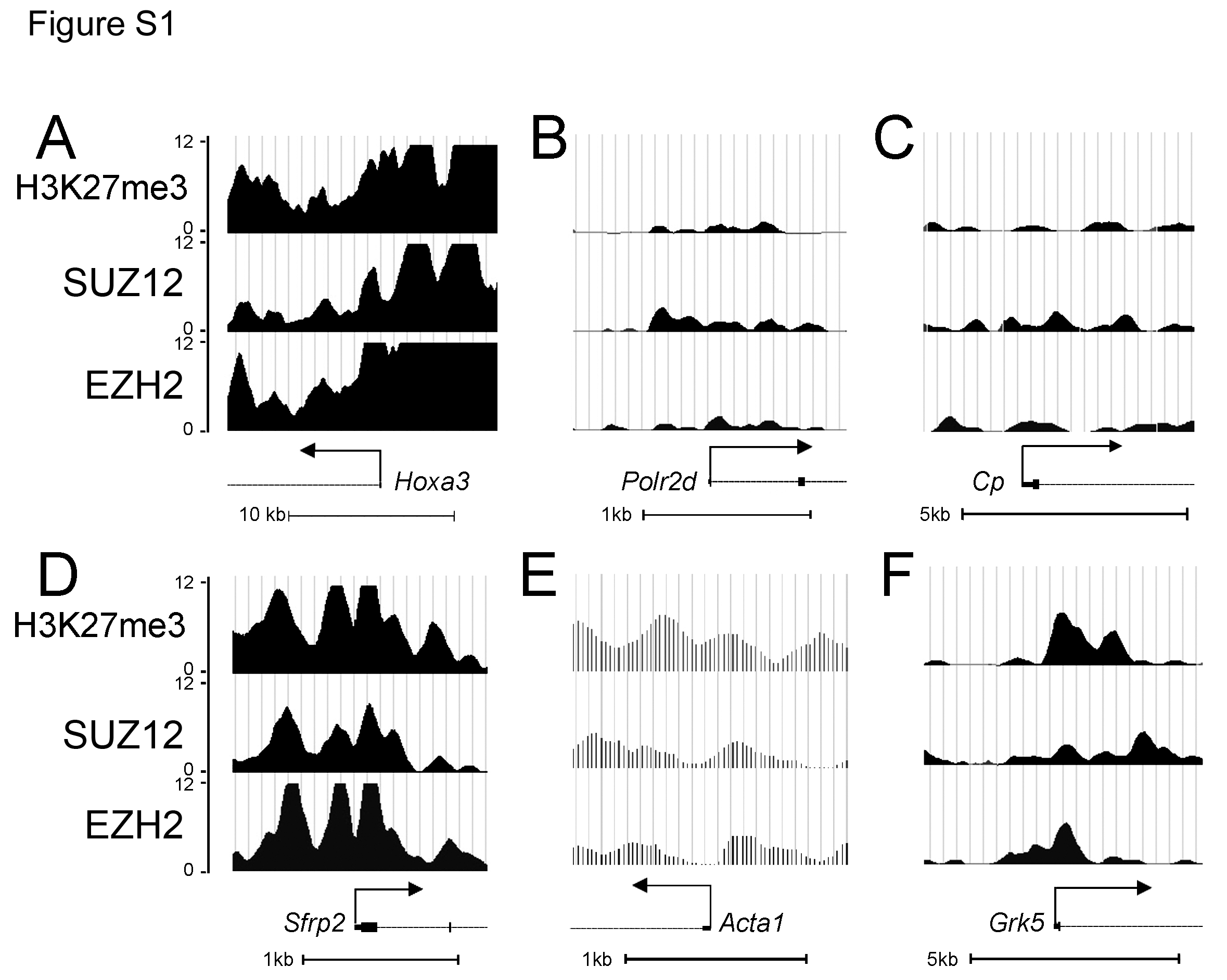

Supplement: Figure S1 — Epigenetic profiles at Sfrp2, Acta1 and Grk5 loci in ES cells. The Broad Institute ChIP-seq database (http://www.broadinstitute.org/scientific-community/science/programs/epigenomics/chip-seqdata) was queried for the enrichment of H3K27me3, SUZ12, and EZH2 at the loci of interest. For each gene, only the genomic region around the TSS is shown. The scale bar for each panel is shown at the bottom of the panel. Arrow points to the direction of transcription. The y axis is the relative level of enrichment. (A–C) Representative epigenetic profiles for three types of genes in ES cells: those that are repressed by PcG activity, those that are constitutively expressed and not regulated by PcG activity, and those that are repressed via PcG-independent mechanism. (A) The chromatin region near the TSS of Hoxa3, a classical PcG target gene, displays high levels of enrichment of H3K27me3, SUZ12 and EZH2. (B) The profile for Polr2d, a housekeeping gene that encodes an RNA polymerase II subunit, shows no enrichment of H3K27me3, SUZ12 or EZH2. (C) H3K27me3 and PRC2 components are not enriched near the TSS of Cp, a gene that is repressed in ES cells. (D–F) The epigenetic profiles around the TSS of Sfrp2, Acta1 and Grk5 resemble that for Hoxa3. (TIF) [file pone.0073983.s002.tif]

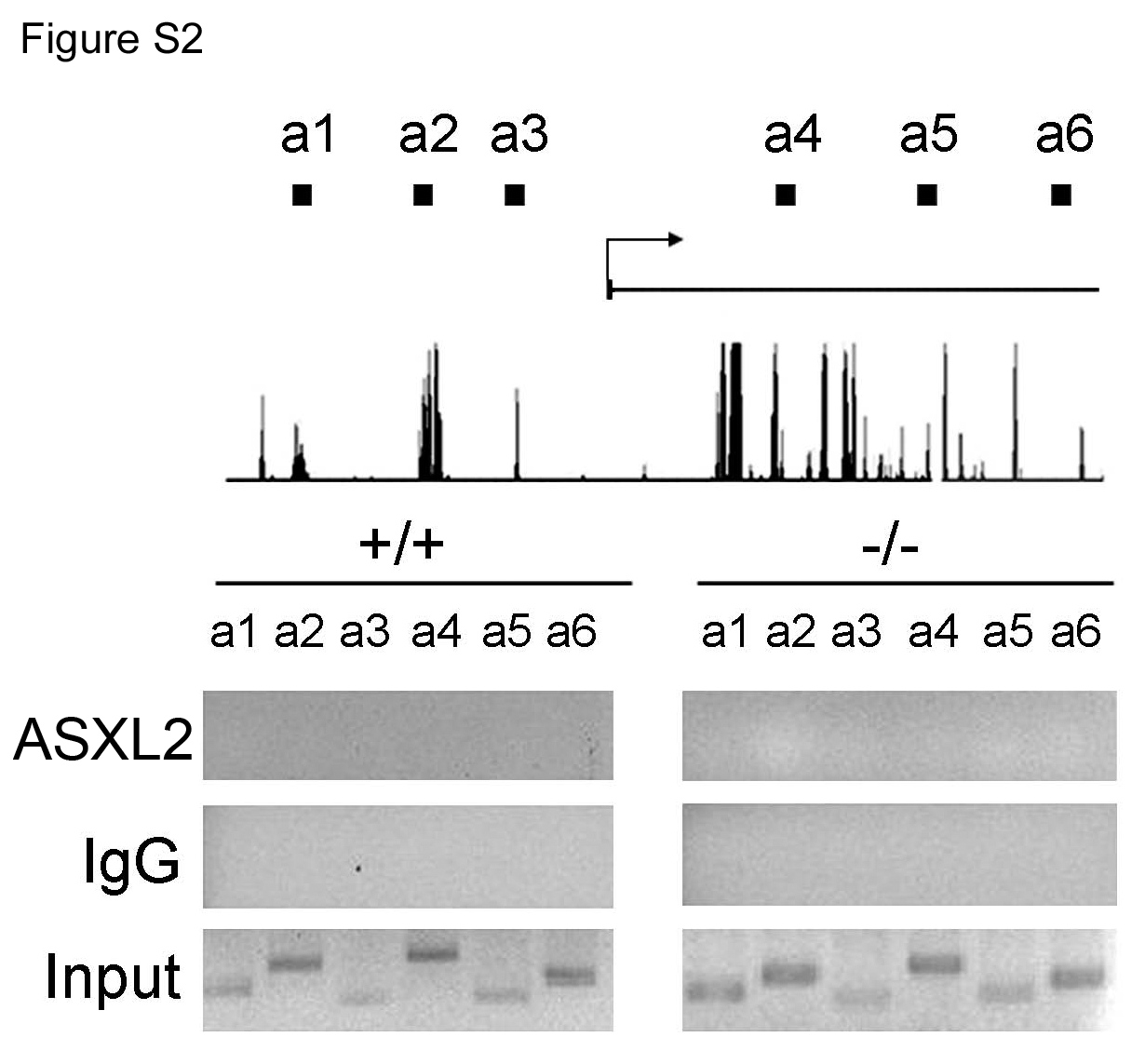

Supplement: Figure S2 — ASXL2 is not enriched at the S100a10 locus. S100a10 encodes a calcium binding protein and is highly expressed in both wild-type and Asxl2 -/- hearts. Shown are anti-ASXL2 ChIP-PCR results for six chromatin sites (a1-a6) within -5kb to +5kb of S100a10 TSS. Mock ChIP was performed with normal rabbit IgG. Input: PCR assay of 1:100 diluted total input chromatin. (TIF) [file pone.0073983.s003.tif]

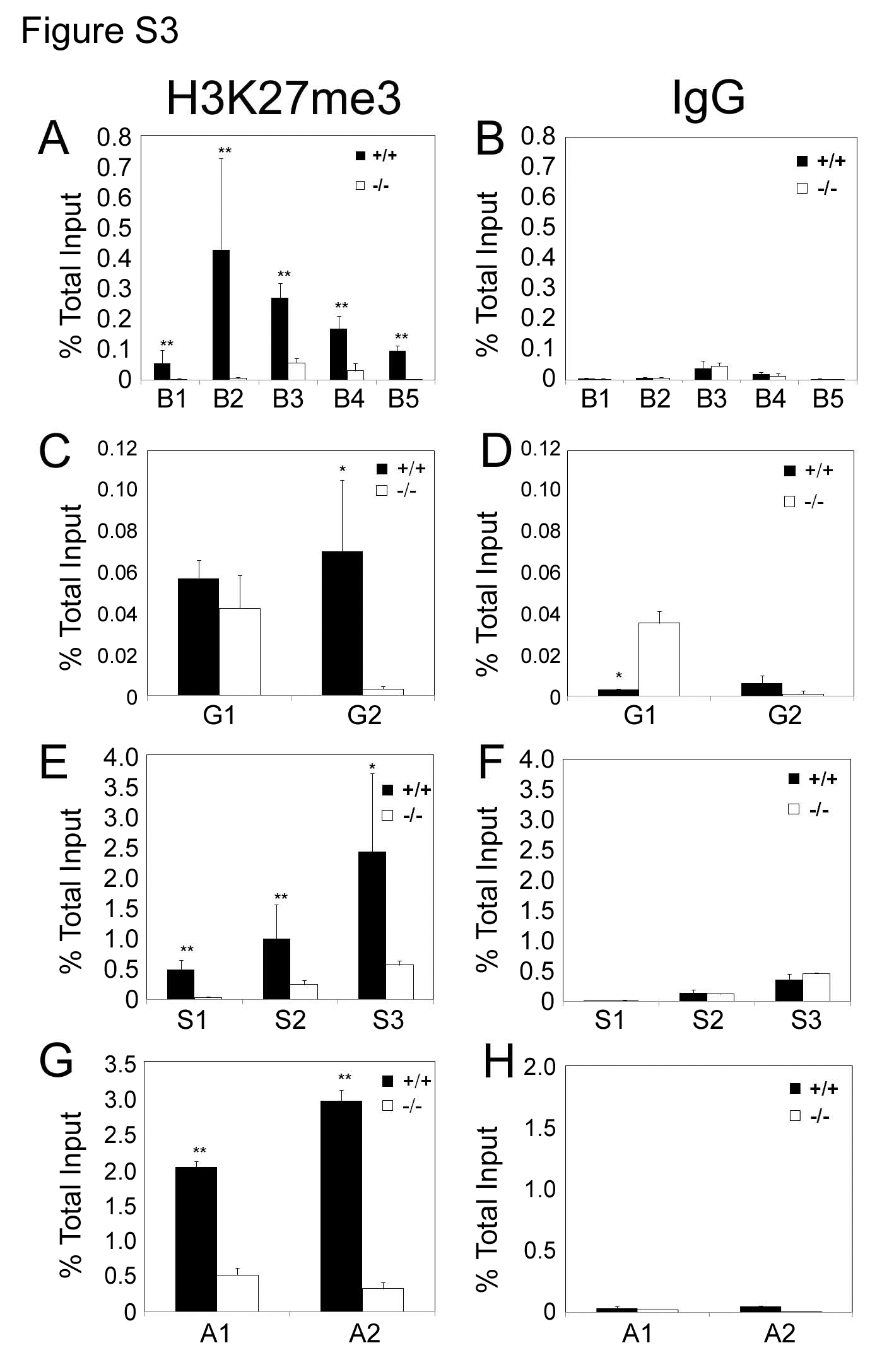

Supplement: Figure S3 — ChIP-qPCR analysis of H3K27me3 enrichment at β-MHC (A–B), Grk5 (C–D), Sfrp2 (E–F) and Acta1 (G–H) loci, shown as percentages of total input. (A, C, E, G) H3K27me3 ChIP. (B, D, F, H) Mock IgG ChIP. Each column represents the mean value of data from three independent samples. *p<0.05; **p<0.01; Error bar: standard deviation. (TIF) [file pone.0073983.s004.tif]

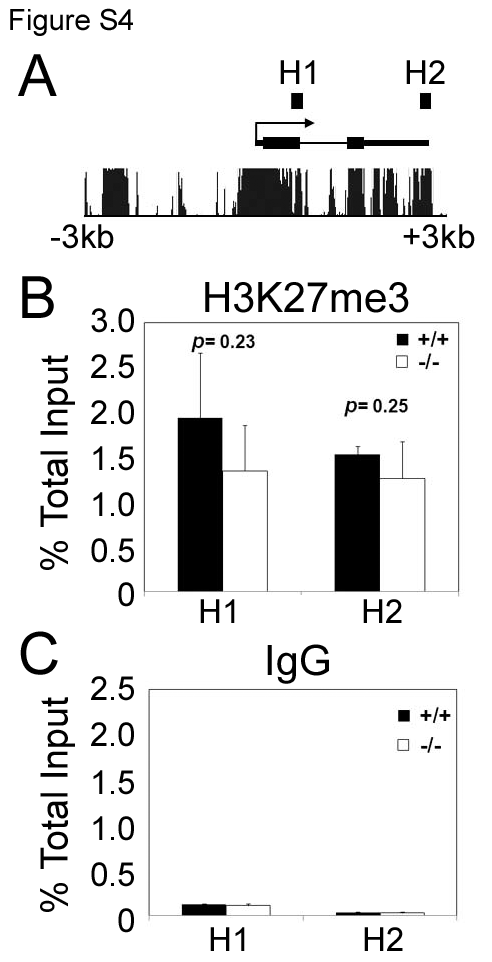

Supplement: Figure S4 — ChIP-qPCR analysis of H3K27me3 enrichment at the Hoxb5 locus, shown as percentages of total input. (A) Alignment of mouse, rat and human genomic sequences from -3kb to +3kb of Hoxb5. H1 and H2 are two highly conserved regions that were selected for ChIP-qPCR analysis. (B) H3K27me3 ChIP. (C) Mock IgG ChIP. Each column represents the mean value of data from three independent samples. Error bar: standard deviation. (TIF) [file pone.0073983.s005.tif]

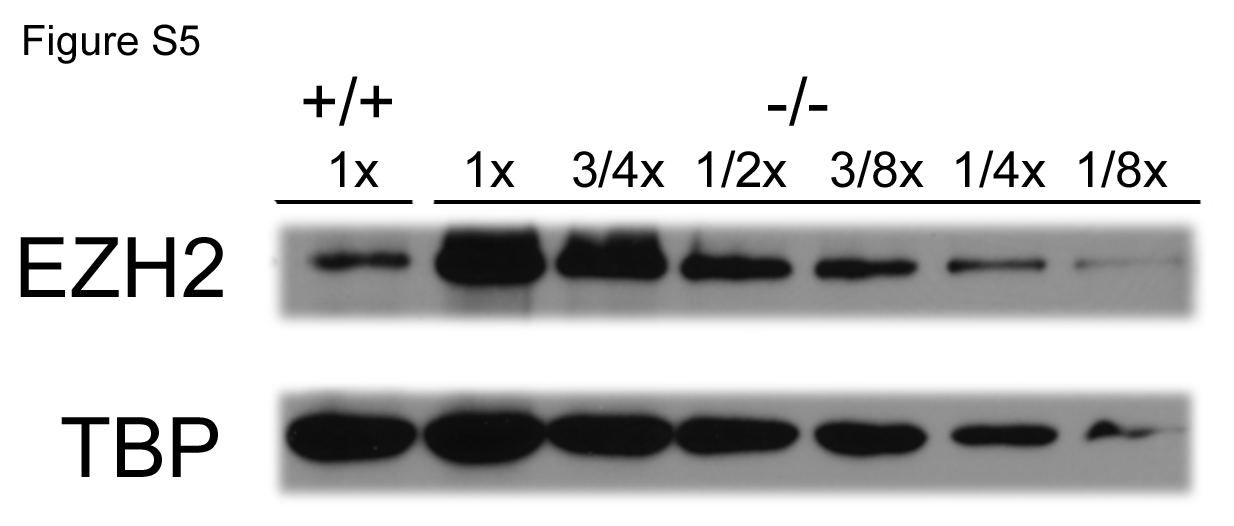

Supplement: Figure S5 — Comparison of EZH2 protein level in wild-type and Asxl2-/- hearts. Serial dilutions of heart extracts were subjected to SDS-PAGE and then probed with anti-EZH2 antibody. Western blot of TBP was used as a loading control. (TIF) [file pone.0073983.s006.tif]

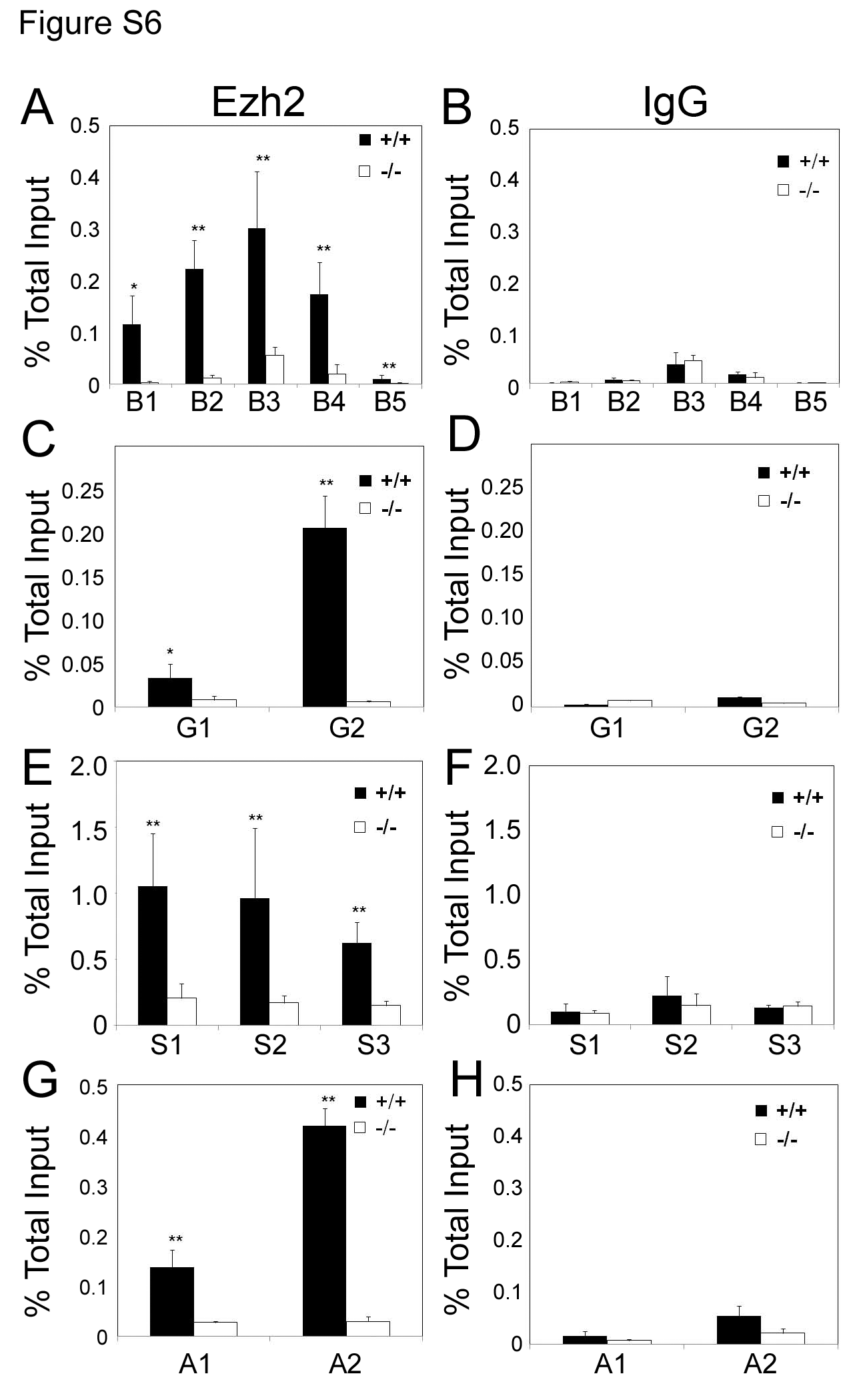

Supplement: Figure S6 — ChIP-qPCR analysis of EZH2 enrichment at β-MHC (A–B), Grk5 (C–D), Sfrp2 (E–F) and Acta1 (G–H) loci, shown as percentages of total input. (A, C, E, G) EZH2 ChIP. (B, D, F, H) Mock IgG ChIP. Each column represents the mean value of data from three independent samples. *p<0.05; **p<0.01; Error bar: standard deviation. (TIF) [file pone.0073983.s007.tif]

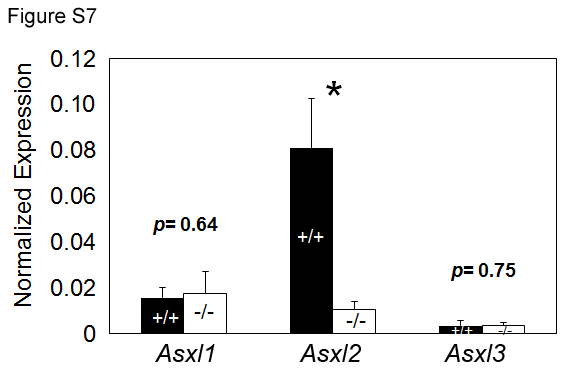

Supplement: Figure S7 — Expression of Asxl genes in the adult mouse heart. The mRNA levels of Asxl1, Asxl2, and Asxl3 in wild-type and Asxl2 -/- hearts were analyzed by real-time RT-PCR. Each column shown is the mean value of data generated from three independent samples. *p<0.05; Error bar: standard deviation. (TIF) [file pone.0073983.s008.tif]
