# Supplementary material for: Additional Sex Combs-Like 2 Is Required for Polycomb Repressive Complex 2 Binding at Select Targets
Source: PLoS One. 2013 Sep 9;8(9):e73983. doi: 10.1371/journal.pone.0073983 (PMC3767597; doi:10.1371/journal.pone.0073983)
Supplement: Methods S1 — Supporting Methods. (DOC) [file pone.0073983.s009.doc]

SUPPLEMENTARY METHODS

ChIP PCR primers
Grk5-1-F (G1F)	AGACAGTTGCAGGGACGAGT	
Grk5-1-R (G1R)	ATTGGCACGTTTTCTTGCTT	
Grk5-2-F (G2F)	GAGGAGAATGGAGTGACAGA	
Grk5-2-R (G2R)	CTTCTTCCTCCCTCTGTGT	
Grk5-3-F (G3F)	AACACGGTCTTGCTGAAAGC	
Grk5-3-R (G3R)	CACTCGCACCAACTCACTGT	
		
Acta1-1-F (A1F)	CCCTTGCACAGGTTTTTA	
Acta1-1-R (A1R)	AAATATGGCTTGGAAGG	
Acta1-2-F (A2F)	CCACGCTCAGTGAGGATTTT	
Acta1-2-R (A2R)	TGCCCATCTATGAGGGCTAT	
		
Sfrp2-1-F (S1F)	CAGCCCGACTTCTCCTACAA	
Sfrp2-1-R (S1R)	AGCCGCATGTTCTGGTACTC	
Sfrp2-2-F (S2F)	CCAGCCCCAGAAAGTAGT	
sfrp2-2-R (S2R)	AATCTGGAGGTGGAGGAG	
sfrp2-3-F (S3F)	GCTTCCTTACCCCTCTCTGG	
Sfrp2-3-R (S3R)	CCTGCCAGGAAAGATTGCTA	
Sfrp2-4-R (S4R)	GACTTTCGTTGCCTCCTCCT	
Sfrp2-4-F (S4F)	AGGCCGGTCACTACTTTCTG	
Sfrp2-5-F (S5F)	TTCGTGATGCCTCTGACAAG	
Sfrp2-5-R (S5R)	GACTGGCTTGAGCAATGTGA	
Sfrp2-6-F (S6F)	TCACATTGCTCAAGCCAGTC	
Sfrp2-6-R (S6R)	CCTGTCCCCGAGTAACCATA	
Sfrp2-7-F (S7F)	CCCACCTTCTGACCTTGGTA	
Sfrp2-7-R (S7R)	CTTGTCAGAGGCATCACGAA	
Sfrp2-8-F (S8F)	TCCCTCTGGGTGTTTACCTG	
Sfrp2-8-R (S8R)	GGATGTTTGCTGCCTGATTT	
		
β-MHC-F1	GGAACCAGCGGAGTACAAAA	
β-MHC-R1	TGTATCCCCTGACCTTGGAG	
β-MHC-F2	CTGCCCCTTTGTCTTGTCTC	
β-MHC-R2	CCCAGGCTTCAGAGTACAGC	
β-MHC-F3	TGCAACTGCATTCTGAGGAC	
β-MHC-R3	ACCAAAGCAGGGGTAAGGAT	
β-MHC-F4	CAACTTCCTATCTGCTGAGG	
β-MHC-R4	GATCCATTTAAGTGCTTTGC	
β-MHC-F5	GGGGAGGATACTGGAAATAG	
β-MHC-R5	GAGAGTAAGCTGACCACGAC	


S100a10-1-F (a1F)	GGGGTTTCTGAGGGTAAAGG	
S100a10-1-R (a1R)	AGAGCACATACGTGGCACTG	
S100a10-2-F (a2F)	GCCTAGCTGGTTGCTGATTC	
S100a10-2-R (a2R)	GGCAGCTCAGACAAGAAAC	
S100a10-3-F (a3F)	AGCTCCTGAAGCTGACAAGC	
S100a10-3-R (a3R)	TGTTGAGTGCAGAACCAAGG	
S100a10-4-F (a4F)	CCTGAGATTTCCTCCACAGC	
S100a10-4-R (a4R)	AATTCTCTATGCGCCACCAC	
S100a10-5-F (a5F)	TGATGTTGTTGGTTGGGTTG	
S100a10-5-R (a5R)	AAAGGAAGCTCCAGATGCAA	
S100a10-6-F (a6F)	CACCCCATCTGGAGTGAAGT	
S100a10-6-R (a6R)	GTCCTAACCAATCCCCCATT	
		
Hoxb5-1-F (Hb5-1F)	TGAGGAAGCTTCACATCAGCCACG	
Hoxb5-1-R (Hb5-1R)	CCAAGCTTTGCTCGCCCCCAC	
Hoxb5-2-F (Hb5-2F)	TGCCAGGCCTGTCTCAGTGATT	
Hoxb5-2-R (Hb5-2R)	ACAAGTAGAGGGCACTGGAGTGG	

RT-PCR primers
Acta1-F	ACCGCTCTTGTGTGTGACAA	
Acta1-R	GGAGTCCTTCTGACCCATACC	
Sfrp2-F	CGTGGGCTCTTCCTCTTCG	
Sfrp2-R	ATGTTCTGGTACTCGATGCCG	
Grk5-F	GGAAGGGGGTGGAGGAAAG	
Grk5-R	AGAACTGTCGAAAAAGCAGTCTC	
Ezh2-F	ATCTGAGAAGGGACCGGTTT	
Ezh2-R	TGTGCACAGGCTGTATCCTC	
